# Manipulation of the Structure and Optoelectronic Properties through Bromine Inclusion in a Layered Lead Bromide Perovskite

**DOI:** 10.1021/acs.chemmater.2c03125

**Published:** 2023-05-03

**Authors:** Lin-jie Yang, Wenye Xuan, David Webster, Lethy Krishnan Jagadamma, Teng Li, David N. Miller, David B. Cordes, Alexandra M. Z. Slawin, Graham A. Turnbull, Ifor D. W. Samuel, Hsin-Yi Tiffany Chen, Philip Lightfoot, Matthew S. Dyer, Julia L. Payne

**Affiliations:** † School of Chemistry, University of St Andrews, North Haugh, St Andrews KY16 9ST, Fife, United Kingdom; ‡ Department of Chemistry, University of Liverpool, Crown Street, Liverpool L69 7ZD, United Kingdom; § Materials Innovation Factory, University of Liverpool, 51 Oxford Street, Liverpool L7 3NY, United Kingdom; ∥ Department of Engineering and System Science, National Tsing Hua University, Hsinchu 30013, Taiwan; ⊥ Organic Semiconductor Centre, School of Physics and Astronomy, University of St Andrews, North Haugh, St Andrews KY16 9SS, Fife, United Kingdom

## Abstract

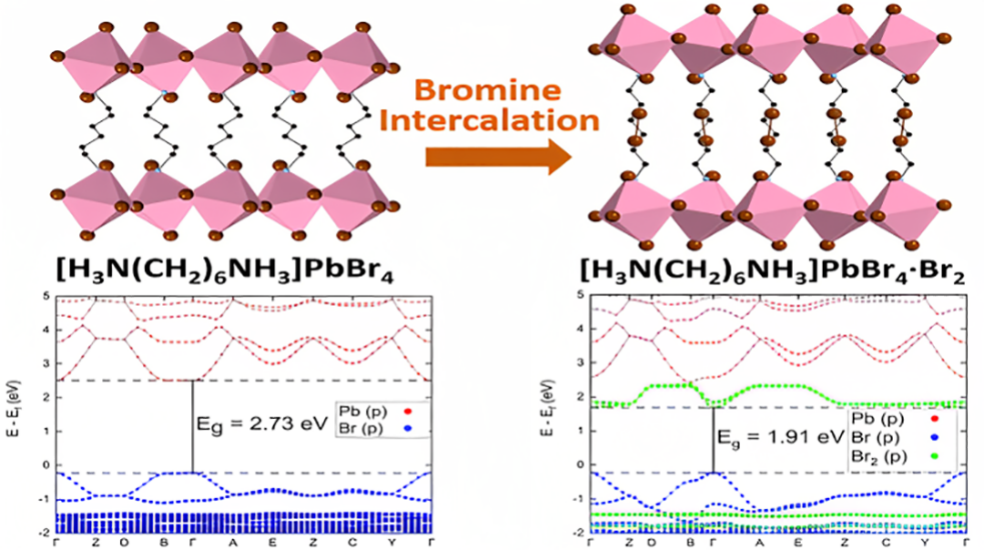

One of the great advantages of organic–inorganic metal halides is that their structures and properties are highly tuneable and this is important when optimizing materials for photovoltaics or other optoelectronic devices. One of the most common and effective ways of tuning the electronic structure is through anion substitution. Here, we report the inclusion of bromine into the layered perovskite [H_3_N(CH_2_)_6_NH_3_]PbBr_4_ to form [H_3_N(CH_2_)_6_NH_3_]PbBr_4_·Br_2_, which contains molecular bromine (Br_2_) intercalated between the layers of corner-sharing PbBr_6_ octahedra. Bromine intercalation in [H_3_N(CH_2_)_6_NH_3_]PbBr_4_·Br_2_ results in a decrease in the band gap of 0.85 eV and induces a structural transition from a Ruddlesden–Popper-like to Dion–Jacobson-like phase, while also changing the conformation of the amine. Electronic structure calculations show that Br_2_ intercalation is accompanied by the formation of a new band in the electronic structure and a significant decrease in the effective masses of around two orders of magnitude. This is backed up by our resistivity measurements that show that [H_3_N(CH_2_)_6_NH_3_]PbBr_4_·Br_2_ has a resistivity value of one order of magnitude lower than [H_3_N(CH_2_)_6_NH_3_]PbBr_4_, suggesting that bromine inclusion significantly increases the mobility and/or carrier concentration in the material. This work highlights the possibility of using molecular inclusion as an alternative tool to tune the electronic properties of layered organic–inorganic perovskites, while also being the first example of molecular bromine inclusion in a layered lead halide perovskite. By using a combination of crystallography and computation, we show that the key to this manipulation of the electronic structure is the formation of halogen bonds between the Br_2_ and Br in the [PbBr_4_]_∞_ layers, which is likely to have important effects in a range of organic–inorganic metal halides.

## 1 Introduction

Organic–inorganic metal halides have received unprecedented attention, which has largely been driven by their applications in solar cells, since the first report of utilizing CH_3_NH_3_PbI_3_ in a photovoltaic device in 2009.^1−3^ They are solution-processable, and over the years, advances have been made in controlling the film morphology, which is essential for producing high-quality devices.^4−7^ The latest certified power conversion efficiencies for CH_3_NH_3_PbI_3_-based photovoltaics are 25.8% for a single junction device and 33.2% for a tandem device made with Si.^8^ Aside from applications in solar cells, organic–inorganic metal halides have been shown to have possible applications in a variety of other devices such as LEDs and sensors.^9−11^

The archetypal inorganic–organic metal halide, CH_3_NH_3_PbI_3_, adopts a perovskite structure (ABX_3_), with CH_3_NH_3_^+^ being disordered on the perovskite A site, Pb^2+^ sitting on the B site, and I^–^ sitting on the X site.^12,13^ Investigations into CH_3_NH_3_PbI_3_ photovoltaics quickly established that the structure could be tuned by isovalent doping on the A, B, or X site.^4,6,14^ As the electronic structure of the valence band and conduction band is dominated by orbital overlap of lead and the halide ligands, halide substitution in particular has been found to have a dramatic effect on tuning the band gap of these semiconductors.^14^ Larger ammonium cations cannot be accommodated in the three-dimensional perovskite structure.^15,16^ Instead, other perovskite-related structures may form.^17,18^ The crystallographic characterization of a large number of layered perovskites has been reported and these layered perovskites can be divided into different categories, such as Ruddlesden–Popper (RP) phases and Dion–Jacobson (DJ) phases, both of which have analogues in oxide perovskites. Several other categories such as those based on diammonium cations and alternating cations in the interlayer spaces (ACI) type have been reported.^19,20^ By the convention adopted in the original inorganic oxides, in the RP phases, [PbI_4_]_∞_ layers adopt a “staggered” conformation with respect to each other, whereas the DJ phases consist of the [PbI_4_]_∞_ layers in an “eclipsed” conformation. In fact, the situation is more complicated than that in hybrid perovskites, and “intermediate” degrees of layer shift are common,^21^ with the organic cation acting like a template and influencing the structural relationship of the neighboring inorganic layers to each other. In turn, this enables the fine tuning of the optoelectronic properties of the material.^22^

Tsai et al. showed that careful control of film deposition for layered perovskites through the use of a hot-casting technique can produce epitaxial thin films of RP phases that exhibit high power conversion efficiencies (12.52%) when incorporated into photovoltaic devices.^23^ These perovskites were found to have extremely high stability to light, humidity, and heat.^23^ Although the number of inorganic layers in RP phases (e.g., (BA)(MA)_*n*−1_Pb_*n*_I_3*n*+1_ (where BA = butyl ammonium and MA = methylammonium) and CH_3_NH_3_^+^) can be changed by adjusting the chemical composition, obtaining phase-pure films with a controlled number of inorganic layers is tricky, as often the final product can be contaminated with layered perovskites that contain fewer inorganic layers in the final product.^24^ Recently, “memory seed effects” have been used to produce high-purity layered perovskite thin films and a power conversion efficiency of 17.1% was reported for the RP phase BA_2_MA_3_Pb_4_I_13_.^24,25^ This memory seed effect was also tested for layered perovskites that adopt the DJ structure type, with power conversion efficiencies of around 14–16% being reported for (4-AMP)MA_3_Pb_4_I_13_ (4-AMP = 4-amino methyl piperidine).^24^ Such high power conversion efficiencies indicate that layered perovskites are also promising absorbers for photovoltaic devices, aside from the more widely studied three-dimensional materials that are based on CH_3_NH_3_PbI_3_.

In order to optimize performance of the layered perovskites in solar cells, it is essential to understand how the crystal structure influences properties. Layered perovskites can be thought of as quantum wells, whereby the organic cation usually takes the role of an insulating, non-conductive spacer. As a result, excitons can be confined in the inorganic [PbX_4_]_∞_ layers. The excitonic binding energy is influenced by the organic cations and for some layered perovskites, the exciton binding energy can be around 200 meV, which is significantly higher than the three-dimensional perovskites.^26−28^ However, studies have shown that these excitons may be readily dissociated into free charge carriers at the edge of the perovskite layers.^26^ Low exciton binding energies are required for efficient charge generation. It is also desirable to determine the effective masses of charge carriers in order to give an insight into charge carrier mobility in new materials. For example, in a recent study, the reduced excitonic effective mass in CH_3_NH_3_PbI_3_ was experimentally determined to be 0.104 m_e_ (where m_e_ is the mass of an electron) that is in agreement with the calculations.^27^ FAPbI_3_ also exhibits a similar effective mass of 0.09 m_e_, while MAPbBr_3_ exhibits an effective mass of 0.117 m_e_ at 2 K.^29^ Mohite et al. studied the exciton reduced masses of (BA)(MA)_*n*_Pb_*n*_I_3*n*+1_ RP phases experimentally and found the exciton reduced masses of 0.221 m_0_ and 0.186 m_0_ for *n* = 1 and 5, respectively.^28^

Intercalation has played a particularly important role in controlling the crystallization kinetics of CH_3_NH_3_PbI_3_ or FAPbI_3_ during thin film preparation through the incorporation of solvent molecules into PbI_2_ thin films, which are precursors in the preparation of particular perovskites.^3,30^ However, the nature of such films has generally prevented single-crystal X-ray diffraction data from being obtained, which would confirm the position of the solvent molecule between the lead iodide layers. In the case of FAPbI_3_, *N*-methyl-2-pyrrolidone (NMP) was found to help stabilize cubic α-FAPbI_3_ to room temperature through the formation of a PbI_2_-NMP compound that was highly strained.^30^ Apart from NMP, dimethyl sulfoxide (DMSO) and *N*,*N*-dimethylformamide (DMF) have also been postulated to insert into PbI_2_.^31,32^

The intercalation of ions or small molecules into layered perovskite oxides has been known for many years.^33−35^ However, the study of intercalation in organic–inorganic metal halide perovskites has been much less explored. In 1986, Maruyama et al. looked at the *in situ* intercalation of 1-chloronaphthalene and dichlorobenzene into (C_9_H_19_NH_3_)_2_PbI_4_ and hexane into (C_10_H_21_NH_3_)_2_CdCl_4_ using powder X-ray diffraction.^33^ The corresponding increases in unit cell parameters were observed, although in-depth crystallographic studies of the intercalation site were not carried out. In 2002, Mitzi et al. intercalated small molecules such as C_6_H_6_ and C_6_F_6_ into [C_6_H_5_C_2_H_2_NH_3_]SnI_4_ to form [C_6_H_5_C_2_H_2_NH_3_]SnI_4_·C_6_H_6_ and [C_6_H_5_C_2_H_2_NH_3_]SnI_4_·C_6_F_6_, respectively.^36^ This resulted in a small change in electronic properties, with an increase of only 0.04 eV in the excitonic peak position upon intercalation of C_6_F_6_.^36^ In this instance, 30 min of heating at 60 °C could remove C_6_F_6_.

More recently, Nag et al. studied iodine intercalation into (BA)_2_PbI_4_, although in-depth single-crystal X-ray diffraction studies were not carried out on the intercalated material.^37^ They found that the PL exhibited two excitonic emissions, but upon intercalation of iodine, only one peak remained.^37^ This was attributed to interactions between adjacent lead iodide layers.^37^ By increasing the length of the carbon chain in the amine, the PL emission could be tuned from dual emission to single emission.^37^ This work was extended by exploring the intercalation of a range of other molecules into different layered perovskites. Hexane and hexafluorobenzene could be intercalated into [C_10_H_12_NH_3_]_2_PbI_4_ and [C_6_H_5_C_2_H_2_NH_3_]SnI_4_, respectively. This resulted in a shift in the absorbance of varying degrees depending on the nature of the molecule and the layered perovskite “host” material and could also tune the PL from dual to single emission.^37^ Ultimately, by careful choice of the spacer amine and intercalation molecule, the PL could be switched from dual to single emission spectra.

Karunadasa et al. reported the intercalation of I_2_ into thin films of (C_6_H_13_NH_3_)_2_PbI_4_ to give (C_6_H_13_NH_3_)_2_PbI_4_·*x*I_2_, although we note that the quantity of iodine intercalated was not stated. They found that iodine deintercalates from a thin film after 10 min, indicating that these materials have limited stability.^38^ In order to improve stability, a closely related, iodide-substituted organic cation, IC_6_H_12_NH_3_^+^, was used to fabricate (IC_6_H_12_NH_3_)_2_PbI_4_.^38^ Iodine intercalation in this material was accompanied by a color change from orange to red, which resulted in an estimated change in band gap from 2.56 to 2.49 eV.^38^ In this case, iodine intercalation also resulted in a decrease in exciton binding energy of 50 meV, with (IC_6_H_12_NH_3_)_2_PbI_4_·I_2_ having the lowest reported exciton binding energy of 180 meV of any *n* = 1 lead iodide perovskite known to date.^38^ The stability of (IC_6_H_12_NH_3_)_2_PbI_4_·I_2_ was found to be four times greater than (C_6_H_13_NH_3_)_2_PbI_4_·I_2_, as I_2_ was retained in the film for longer at room temperature.^38^ Although the examples above relate to intercalation of molecules into layered perovskites, we note that there are a few examples of halogenmolecule incorporation into halide-based compounds that do not adopt a perovskite structure.^39,40^

The importance of hydrogen bonds in organic–inorganic metal halides is well known and has been documented by others. However, another type of non-covalent interaction is the halogen bond. Hassel crystallographically characterized the first halogen bonds and was awarded the Nobel Prize in 1969.^41^ Although the first experimental evidence for halogen bonds was reported in the 1800s, IUPAC only formally issued a recommendation for the definition of the halogen bond in 2013.^42,43^ The halogen bond, R–X···B, is a non-covalent interaction between a covalently bonded halogen (X) and an electron-rich species (B). The covalently bound halogen atom has a region called a sigma hole that is electron-poor and hence has a slight positive charge, which extends along the R–X axis. This bonds to a nucleophilic species that may be negatively charged and could come from the same molecular species.^44^

To date, there have been several studies that exploit the use of halogen bonding at the interfaces of materials in solar cells that utilize inorganic–organic metal halides.^45,46^ This is beneficial as it passivates the interface.^45,46^ Halogen bonding can enhance a variety of photovoltaic parameters, for example, by enhancing carrier lifetime by reducing recombination due to improved crystallization.^47^ It can improve the stability of the materials toward moisture and can modulate crystallization kinetics to get the desired film morphology and grain size.^48^ However, to date, little attention has been given to how halogen bonding influences the bulk perovskite crystal structure. Several examples of compounds that could exhibit halogen bonding have been reported but this has not been studied in detail and can often be overlooked. Here, in a combined experimental–theoretical study, we show that intercalation of bromine molecules in a Ruddlesden–Popper perovskite [H_3_N(CH_2_)_6_NH_3_]PbBr_4_ can manipulate its structure, electronic structure, and optoelectronic properties. The key to this manipulation is the formation of halogen bonds between the bromine molecule and the [PbBr_4_]_∞_ layers.

## 2 Experimental Section

### 2.1 Starting Materials

1,6-Diaminohexane (H_2_N(CH_2_)_6_NH_2_, ≥98%), lead(II) bromide (PbBr_2_, ≥98%), bromine liquid (Br_2_, 99.8%), and hydrobromic acid (HBr, 48%, w/w aqueous solution) were purchased from Alfa Aesar. All chemicals were directly used without further purification.

### 2.2 Single-Crystal Growth

#### 2.2.1 [H_3_N(CH_2_)_6_NH_3_]PbBr_4_

PbBr_2_ (0.714 g, 2 mmol) was dissolved in concentrated HBr (8 mL) with moderate heating and stirring. Once the PbBr_2_ had dissolved, H_2_N(CH_2_)_6_NH_2_ (0.25 g, 2.15 mmol) was added into the warm mixture. The temperature of this mixture was increased to 90 °C, with vigorous stirring, until all precipitates disappeared. The resulting colorless/pale-yellow solution was kept at 50 °C for 24 h so that most of the product would form as colorless chip-shaped crystals.

#### 2.2.2 [H_3_N(CH_2_)_6_NH_3_]PbBr_4_·Br_2_

PbBr_2_ (0.714 g, 2 mmol), H_2_N(CH_2_)_6_NH_2_ (0.25 g, 2.15 mmol), HBr (5 mL), and Br_2_ (5 mL) were sealed in a 30 mL Teflon-lined stainless steel autoclave. The autoclave was placed in an oven at 160 °C for 8 h and this was followed by a further 24 h in the oven at 80 °C. The autoclave was cooled naturally to room temperature and orange chip-shaped crystals were obtained.

### 2.3 Polycrystalline Sample Preparation

#### 2.3.1 [H_3_N(CH_2_)_6_NH_3_]PbBr_4_

PbBr_2_ (0.714 g, 2 mmol) was dissolved in concentrated HBr (8 mL) with moderate heating and stirring. Once the PbBr_2_ had dissolved, H_2_N(CH_2_)_6_NH_2_ (0.25 g, 2.15 mmol) was added into the warm solution. The temperature of the mixture was increased to 90 °C, with vigorous stirring, until all precipitates disappeared. After the solution was cooled naturally to room temperature, the resulting product consisted of small single crystals. The product was filtered, dried, and then ground into a fine powder.

#### 2.3.2 [H_3_N(CH_2_)_6_NH_3_]PbBr_4_·Br_2_

Br_2_ liquid (5 mL) was poured into a 30 mL Teflon liner of an autoclave. Separately, the white powder of [H_3_N(CH_2_)_6_NH_3_]PbBr_4_ (2.5 g, 3.8 mmol) was placed in a 25 mL glass sample vial and spread evenly across the base of the vial. The sample vial was then placed in the Teflon liner and the Teflon liner would be covered and sealed by Parafilm for 5 days to provide an environment that could have sufficient contact between the Br_2_ vapor and [H_3_N(CH_2_)_6_NH_3_]PbBr_4_ powder. A bright orange powder was obtained when the vessel was opened.

### 2.4 Deintercalation Process

[H_3_N(CH_2_)_6_NH_3_]PbBr_4_·Br_2_ was placed in a glass sample vial on a hotplate at 75 °C for 3 h. Bromine was slowly released (as brown gas) and the powder turned white. The resulting white powder was characterized by PXRD, which indicated that it was phase 3 of [H_3_N(CH_2_)_6_NH_3_]PbBr_4_.

### 2.5 Reintercalation Process

The polycrystalline, deintercalated [H_3_N(CH_2_)_6_NH_3_]PbBr_4_ sample was placed in a 25 mL glass sample vial and distributed evenly across the base of the vial. The sample vial was then placed inside a Teflon autoclave liner with 5 mL of Br_2_ liquid. This was closed with the lid and sealed with Parafilm. The resulting reaction mixture was left for 5 days to provide an environment that could have sufficient contact between the Br_2_ vapor and the [H_3_N(CH_2_)_6_NH_3_]PbBr_4_ powder. An orange powder was obtained when the vessel was opened. PXRD was used to confirm that the powder was [H_3_N(CH_2_)_6_NH_3_]PbBr_4_·Br_2_.

## 3 Characterization

### 3.1 X-ray Diffraction

Single-crystal X-ray diffraction data for [H_3_N(CH_2_)_6_NH_3_]PbBr_4_·Br_2_ were collected at low temperature (LT, 173 K) and room temperature (RT, 298 K) on a Rigaku SCX Mini diffractometer using Mo Kα radiation. Data for [H_3_N(CH_2_)_6_NH_3_]PbBr_4_ were collected at low temperatures (phase 1, 238 K and phase 2, 93 K) and room temperature (phase 3, RT, 298 K) on a Rigaku FR-X Ultrahigh Brilliance Microfocus RA generator/confocal optics with a XtaLAB P200 diffractometer. Data were collected using *CrystalClear* (Rigaku) software.^49^ Structures were solved by direct methods using *SHELXT*, and full-matrix least-squares refinements on *F*^2^ were carried out using *SHELXL*-2018/3 incorporated in the WinGX program.^50−52^ Absorption corrections were performed empirically from equivalent reflections based on multiscans using either *CrystalClear* or *CrysAlisPro* (Rigaku).^53^ Non-H atoms were refined anisotropically and hydrogen atoms were treated as riding atoms. Both the RT and the 93 K data from [H_3_N(CH_2_)_6_NH_3_]PbBr_4_ showed inversion twinning. Data were also collected on [H_3_N(CH_2_)_6_NH_3_]PbBr_4_ at 173 K; however, an isostructural unit cell was found for the data, and a better refinement was obtained from the 238 K data.

Ambient temperature powder X-ray diffraction data were collected on a PANalytical Empyrean diffractometer using Cu Kα_1_ (λ = 1.5406 Å) radiation in the range of 2θ = 3–70°, with a step size of 0.017° and a time per step of 0.913 s.

For variable-temperature powder X-ray diffraction (VT-PXRD), [H_3_N(CH_2_)_6_NH_3_]PbBr_4_ was loaded into a 0.3 mm diameter glass capillary and data were collected on a STOE STADIP diffractometer operating in Debye–Scherrer geometry using Mo Kα_1_ (λ = 0.71075 Å) radiation and a Mythen 2 K detector. Data were collected in the range of 1.5–20° for 120 min. Data are collected every 10 °C from 20 to −70 °C upon cooling and heating.

### 3.2 UV–Visible Spectroscopy

Diffuse reflectance UV–visible spectra were collected on polycrystalline powders of both [H_3_N(CH_2_)_6_NH_3_]PbBr_4_ and [H_3_N(CH_2_)_6_NH_3_]PbBr_4_·Br_2_ using a JASCO-V650 ultraviolet–visible spectrophotometer with a wavelength range of 190–900 nm. BaSO_4_ was used as a reference.

### 3.3 Scanning Electron Microscopy

Scanning electron microscopy was carried out on a JEOL-IT200 equipped with a 25 mm^2^ JEOL DrySD EDS detector and a JEOL JSM 5600.

### 3.4 Raman Spectroscopy

Raman spectroscopy was carried out on a Renishaw in-Via Qontor microsope using a 532 nm laser.

### 3.5 Electrical Measurements

Electrical measurements were carried out on [H_3_N(CH_2_)_6_NH_3_]PbBr_4_ and [H_3_N(CH_2_)_6_NH_3_]PbBr_4_·Br_2_ to measure the mobility values using the space charge limited current (SCLC) method. To carry out these measurements, pellets of [H_3_N(CH_2_)_6_NH_3_]PbBr_4_ and [H_3_N(CH_2_)_6_NH_3_]PbBr_4_·Br_2_ samples were made (13 mm in diameter and thickness varying from 0.7 to 2 mm). The current–voltage measurements were carried out using the all-in-one characterization platform Paios, Fluxim AG, Switzerland. The voltage scan range used was 0–9 V. The contact electrodes were made by aluminum tape, silver paint, and carbon tape. Even though a trap-free space charge limited the current region with *J* ∝ *V*^2^ (where *J* is the current density and *V* is the applied bias) was observed for the [H_3_N(CH_2_)_6_NH_3_]PbBr_4_ sample, such a current regime with *J* ∝ *V*^2^ was not observed for the [H_3_N(CH_2_)_6_NH_3_]PbBr_4_·Br_2_ sample. This observation was consistent for different electrode materials selected. This lack of a clear space charge region in the [H_3_N(CH_2_)_6_NH_3_]PbBr_4_·Br_2_ sample indicated the higher conductivity of these samples. This has been further verified using the resistivity analysis of the samples in the Ohm’s law region.

### 3.6 Photoemission and Kelvin Probe Measurements

Measurements of contact potential difference (CPD) and surface photovoltage spectroscopy (SPS) were made using an SKP5050 Scanning Kelvin Probe, with an attached Fiber-Lite DC 950 QTH white light source. The CPD data, measured relative to the Kelvin probe tip, was converted to an absolute value for the Fermi level by comparison with a gold reference sample of a known work function of 4.8 eV. SPS measurements show the change in surface Fermi level under optical illumination. Light from a QTH lamp was passed through a linear variable filter and directed onto the sample. The CPD was measured while the illumination wavelength was scanned from 1000 to 400 nm. In the SPS measurement, the baseline was determined by averaging the data between 800 and 1000 nm; this baseline was then subtracted from all datapoints to show only the change in CPD due to illumination.

Ambient photoemission spectroscopy (APS) measurements were made using the APS02 module attached to the Kelvin probe system, which comprises a deuterium (D_2_) lamp passed through a monochromator. The incident photon energy was scanned from 4.5 to 7 eV and the resulting photoemission yield was measured as a current at the Kelvin probe tip. Following Fowler theory, the ionization potential of each sample was determined by first subtracting the low-energy baseline from the measured photoemission signal and then taking the cube-root. An extrapolation of the linear region of the resulting graph then allows the ionization potential to be obtained.

### 3.7 Electronic Structure Calculations

Density functional theory (DFT) calculations were applied to [H_3_N(CH_2_)_6_NH_3_]PbBr_4_ and [H_3_N(CH_2_)_6_NH_3_]PbBr_4_·Br_2_ using the Vienna Ab Initio Simulation Package (VASP)^54^ with the projected augmented wave (PAW)^55^ potentials. The hybrid functional (HSE06)^56^ was used throughout with spin–orbital coupling (SOC) effects included except for the calculations of the electron localization function (ELF)^57^ (VASP does not support ELF calculations with SOC). The valence space for Pb, Br, N, C, and H atoms consisted of 14, 7, 5, 4, and 1 electrons, respectively. The energy cutoff was set at 520 eV with a 2 × 3 × 3 Γ center k-point mesh. Since van der Waals (vdW) forces play an important role in the layered hybrid perovskites, the DFT-D3 vdW correction^58^ was applied during every step of the calculation.

First, the crystal structures determined from single-crystal X-ray diffraction data were fully relaxed at a force level of 0.02 eV/Å by the PBE functional.^59^ The crystal structure of phase 1 was used as a starting point for the parent phase, [H_3_N(CH_2_)_6_NH_3_]PbBr_4_. The electronic structures of fully relaxed [H_3_N(CH_2_)_6_NH_3_]PbBr_4_ and [H_3_N(CH_2_)_6_NH_3_]PbBr_4_·Br_2_ were then plotted. To further understand the interaction between Br_2_ and the ELF of the Pb–Br–Br_2_ atom chain in [H_3_N(CH_2_)_6_NH_3_]PbBr_4_·Br_2_ was calculated. To look at the halogen bonding effect, the electron density difference map of [H_3_N(CH_2_)_6_NH_3_]PbBr_4_·Br_2_ was calculated by separating the intercalated structure into two parts, Br_2_ and [H_3_N(CH_2_)_6_NH_3_]PbBr_4_, without changing the position of ions.

As for the mobility properties, hole and electron effective masses of [H_3_N(CH_2_)_6_NH_3_]PbBr_4_ and [H_3_N(CH_2_)_6_NH_3_]PbBr_4_·Br_2_ were calculated through the *x*, *y*, and *z* directions of the lattice with seven numerical points in a width of 0.03 for fitting.

## 4 Results and Discussion

### 4.1 Crystal Structures of [H_3_N(CH_2_)_6_NH_3_]PbBr_4_ and [H_3_N(CH_2_)_6_NH_3_]PbBr_4_·Br_2_

The crystal structures of [H_3_N(CH_2_)_6_NH_3_]PbBr_4_ and [H_3_N(CH_2_)_6_NH_3_]PbBr_4_·Br_2_ were determined from single-crystal X-ray diffraction data at 173 and 298 K. [H_3_N(CH_2_)_6_NH_3_]PbBr_4_ was found to display a complex polymorphism, with three different phases observed in the 93–298 K region. These have been designated phase 1, phase 2, and phase 3. At 298 K, a mixture of two different phases of [H_3_N(CH_2_)_6_NH_3_]PbBr_4_ was found. The simplest of these (now designated phase 1, see Table 1 and Figure S1) was only found as a minority phase in single crystalline form. The majority phase at 298 K (phase 3; see Table S1 and Figure S3) has a much more complex structure, characterized by “undulating” [PbBr_4_]_∞_ layers and resulting in a unit cell four times the volume of phase 1. Phase 1 was found cleanly in our single-crystal data at 238 K, and this phase was also identified in data collected at 173 K. Below this temperature, a further phase (phase 2; see Table S1 and Figure S2) was also found. Phase 2 also exhibits a slight undulation of the [PbBr_4_]_∞_ layers, although this is weaker than that in phase 3 and results in a supercell three times the volume of phase 1.

**Table 1:** Crystallographic Parameters and Refinement Details for [H_3_N(CH_2_)_6_NH_3_]PbBr_4_·Br_2_ and [H_3_N(CH_2_)_6_NH_3_]PbBr_4_

sample	[H_3_N(CH_2_)_6_NH_3_]PbBr_4_·Br_2_ LT	[H_3_N(CH_2_)_6_NH_3_]PbBr_4_·Br_2_ RT	[H_3_N(CH_2_)_6_NH_3_]PbBr_4_ phase 1
CCDC code	2203746	2203747	2203748
formula	Pb_1_Br_6_C_6_N_2_H_18_	Pb_1_Br_6_C_6_N_2_H_18_	PbBr_4_C_6_N_2_H_18_
formula weight	804.87	804.87	645.05
crystal description	orange block	orange prism	colorless prism
crystal size (mm^3^)	0.57 × 0.54 × 0.48	0.14 × 0.13 × 0.11	0.08 × 0.05 × 0.02
temperature (K)	173	298	238
crystal system	monoclinic	monoclinic	monoclinic
space group	*P*2_1_/*c*	*P*2_1_/*c*	*P*2_1_/c
*a* (Å)	14.1357(9)	14.1566(9)	11.8422(4)
*b* (Å)	8.1227(5)	8.1672(5)	8.0378(2)
*c* (Å)	7.9302(5)	8.0035(5)	8.4396(3)
β (deg)	94.022(4)	93.7760(10)	107.746(4)
volume (Å^3^)	908.30(10)	923.35(10)	765.10(5)
*Z*	2	2	2
ρ (calc, g/cm^3^)	2.943	2.895	2.800
μ (mm^–1^)	22.464	22.098	21.440
*F*(000)	720	720	580
reflections collected	8961	9220	9668
independent reflections (*R*_int_)	2079 (0.0608)	2122 (0.0666)	1798 (0.0198)
parameters, restraints	72,0	71, 0	62, 0
goodness-of-fit on *F*^2^	0.992	1.004	1.049
*R*_1_	0.0427	0.0550	0.0232
*R*_1_ [*I* > 2σ(*I*)]	0.0369	0.0355	0.0194
w*R*_2_	0.0941	0.0911	0.0500
w*R*_2_ [*I* > σ(*I*)]	0.0927	0.0840	0.0489
largest diff. peak and hole (e/Å^3^)	3.215 and −3.801	0.996 and −1.880	1.457 and −0.576

**Figure 1:**
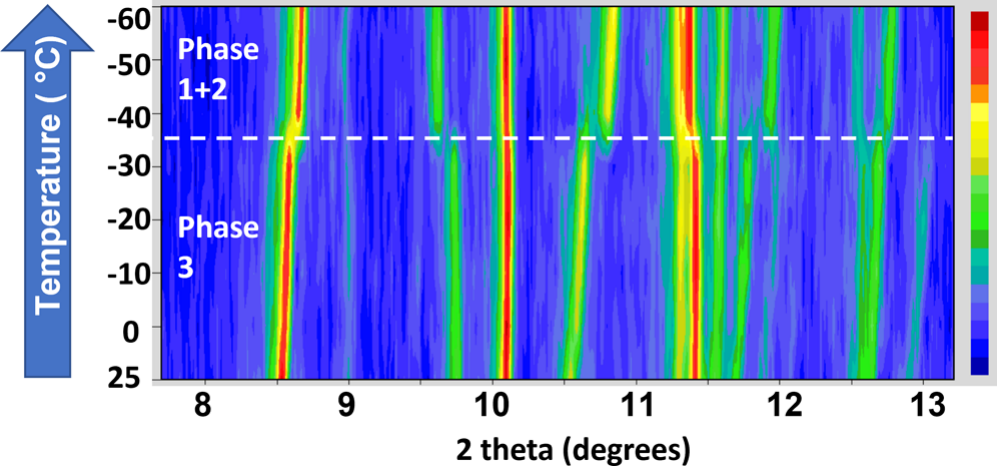
Contour plot of VT-PXRD data collected on [H_3_N(CH_2_)_6_NH_3_]PbBr_4_ between room temperature and −60 °C. Note that data were collected using Mo Kα_1_ radiation and the *y*-axis is not linear.

**Figure 2:**
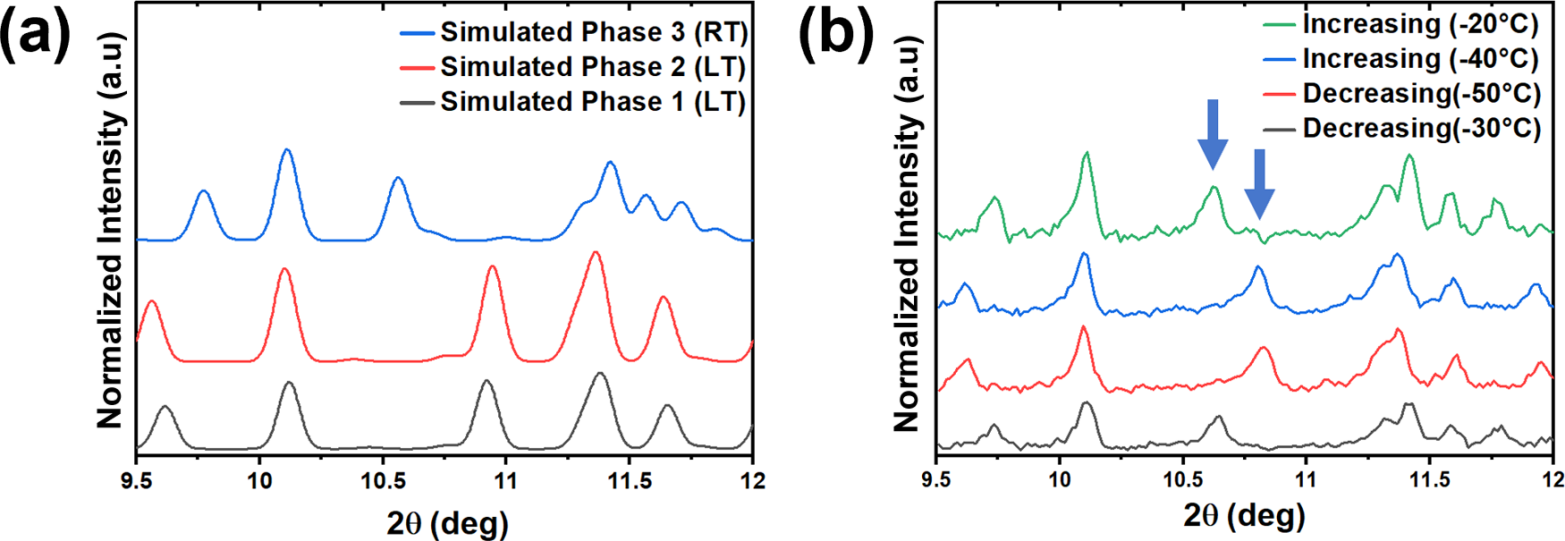
(a) Simulated PXRD patterns and (b) variable-temperature PXRD patterns for [H_3_N(CH_2_)_6_NH_3_]PbBr_4_. Note that as Mo Kα_1_ radiation was used for the variable-temperature experiment, the simulated patterns also are for Mo Kα_1_ radiation. Arrows are used to indicate the peaks in phase 1/2 and phase 3 that are most influenced by the phase transition.

In order to probe the sequence of phase transitions in [H_3_N(CH_2_)_6_NH_3_]PbBr_4_, a variable-temperature PXRD (VT-PXRD) experiment was carried out. A contour plot of a small section of the VT-PXRD data is shown in Figure 1. Clear changes were observed in the −30 to −40 °C region, indicating that a phase transition had occurred. A small portion of the simulated PXRD of the three different phases of [H_3_N(CH_2_)_6_NH_3_]PbBr_4_ (which were determined by single-crystal X-ray diffraction) have been compared in Figure 2a, alongside the VT-PXRD data in the 9.5–12.0 2θ region. Phase 3 was clearly distinguishable from phases 1 and 2 due to its large displacements (undulations) of the lead atoms in the structure (Figures S1–S3). This manifests itself in the PXRD patterns in the 9.5–12.0° 2θ region by the peaks at 10.5 and 10.8°. The VT-PXRD data (Figure 2b) show that phase 3 of [H_3_N(CH_2_)_6_NH_3_]PbBr_4_ was present as the dominant phase at room temperature. Upon cooling, the structure of [H_3_N(CH_2_)_6_NH_3_]PbBr_4_ changed from phase 3 to a mixture of phases 1 and 2 between −30 and −50 °C. As phases 1 and 2 showed extremely similar PXRD patterns at low temperature, the sample could only be described as a mixture of phases 1 and 2, without reliable phase fractions. Upon heating, the phase transition occurred between −40 and −20 °C, and above this temperature, [H_3_N(CH_2_)_6_NH_3_]PbBr_4_ readopted the phase 3 structure, indicating that the phase transformation was reversible. For simplicity, most of the remaining discussion herein is focused on a comparison of phase 1 and its intercalated product [H_3_N(CH_2_)_6_NH_3_]PbBr_4_·Br_2_. [H_3_N(CH_2_)_6_NH_3_]PbBr_4_·Br_2_ can be seen (Table 1) to retain essentially the same structure as phase 1, at both 173 and 298 K, but with a significant expansion of the unit cell parameter *a*, perpendicular to the [PbBr_4_]_∞_ layers together with a significant change in the β angle of the monoclinic unit cell.

Further crystallographic and refinement details are shown in Table 1 and the crystallographic and refinement details for phases 2 and 3 are shown in Table S1, while images of phases 3, 2, and 1 are shown in Figures S1–S3. SEM images of [H_3_N(CH_2_)_6_NH_3_]PbBr_4_ and [H_3_N(CH_2_)_6_NH_3_]PbBr_4_·Br_2_ are shown in Figures S4 and S5 and the Rietveld fit of VT-PXRD data is shown in Figures S6 and S7.

**Figure 3:**
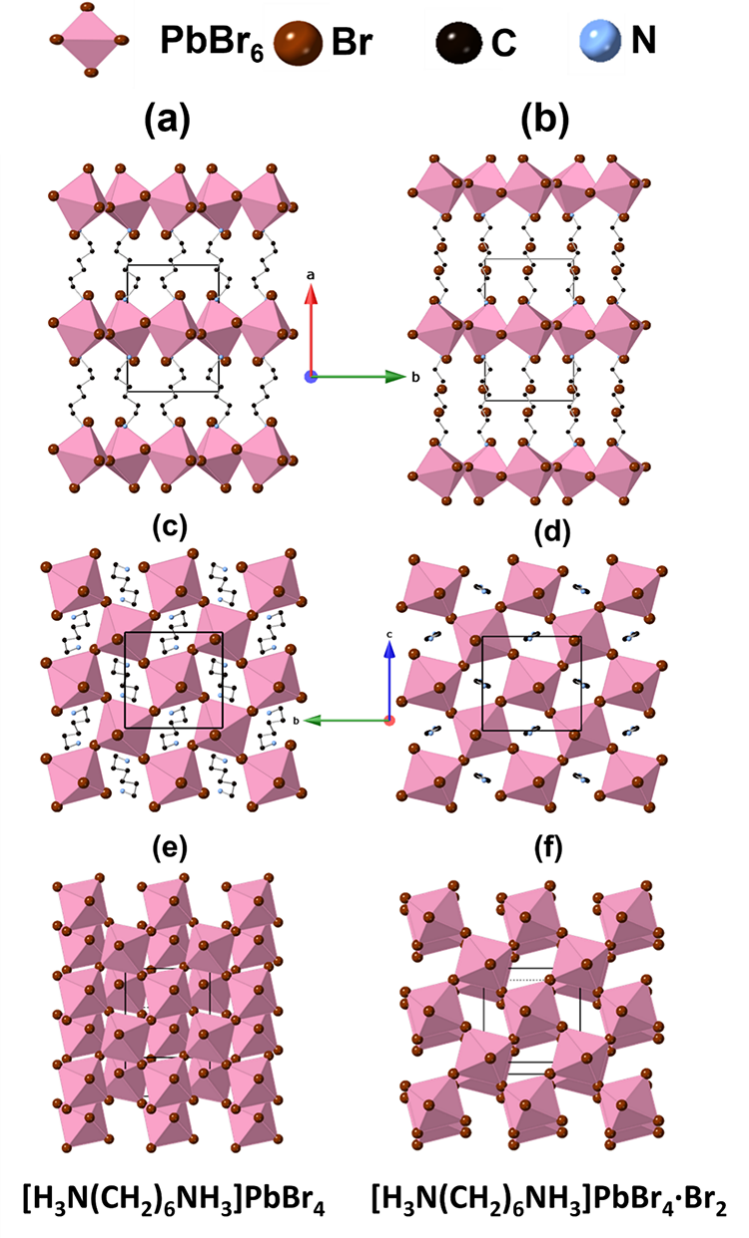
Crystal structures of both (a, c, e) [H_3_N(CH_2_)_6_NH_3_]PbBr_4_ (phase 1) and (b, d, f) [H_3_N(CH_2_)_6_NH_3_]PbBr_4_·Br_2_. (a, b) Projections along the *c*-axis to show the changes upon bromine intercalation, including the expansion along the *a*-axis upon intercalation, (c, d) projections along the *a*-axis to illustrate interlayer shift, and (e, f) vertical projection of the inorganic layers to illustrate the change in conformation of the organic cation. Note that the H_3_N(CH_2_)_6_NH_3_^2+^ cations have been excluded for clarity in panels (c) and (d).

Our single-crystal X-ray diffraction studies show that both [H_3_N(CH_2_)_6_NH_3_]PbBr_4_ and [H_3_N(CH_2_)_6_NH_3_]PbBr_4_·Br_2_ adopt a structure (Figure 3a,b) consisting of single [PbBr_4_]_∞_ layers separated by the [H_3_N(CH_2_)_6_NH_3_]^2+^ cations. This *P*2_1_/*c* structure type is the most common amongst layered hybrid perovskites of this family.^21^ The unit cell can be described as a √2 × √2 × 1 supercell of the aristotype DJ phase. This can be rationalized based on the tilts/rotation of the octahedral units within the [PbBr_4_]_∞_ layers, described by the Glazer-like notation *a*^–^*a*^–^*c*. We note that the more complex phases of [H_3_N(CH_2_)_6_NH_3_]PbBr_4_ retain this same basic tilt system, but differ in displaying a slight undulation of the [PbBr_4_]_∞_ layers (Figures S2 and S3). We can provide no explanation for the adoption of these unusual structures here, but similar “rippled” layers have been noted in other similar systems.^21,60^ The clearest crystallographic difference between the parent perovskite ([H_3_N(CH_2_)_6_NH_3_]PbBr_4_) and [H_3_N(CH_2_)_6_NH_3_]PbBr_4_·Br_2_ at low temperature (LT) is the change in unit cell parameters, especially the expanded *a*-axis and the reduced angle β. The expanded *a*-axis clearly correlates with the incorporation of the Br_2_ moiety, shown in Figure 3 and discussed in more detail below. The change in β correlates with the smaller degree of layer shift^21^ in [H_3_N(CH_2_)_6_NH_3_]PbBr_4_·Br_2_ (viz. (0.13, 0.13) versus (0.39,0.39)), as shown in the projection onto the [PbBr_4_]_∞_ layers in Figure 3e,f. Hence, there is a change from nRP to nDJ type upon intercalation. Again, this is ultimately driven by the incorporation of Br_2_ and subsequent modifications to the conformation of the amine and its interaction with the inorganic layers.

**Figure 4:**
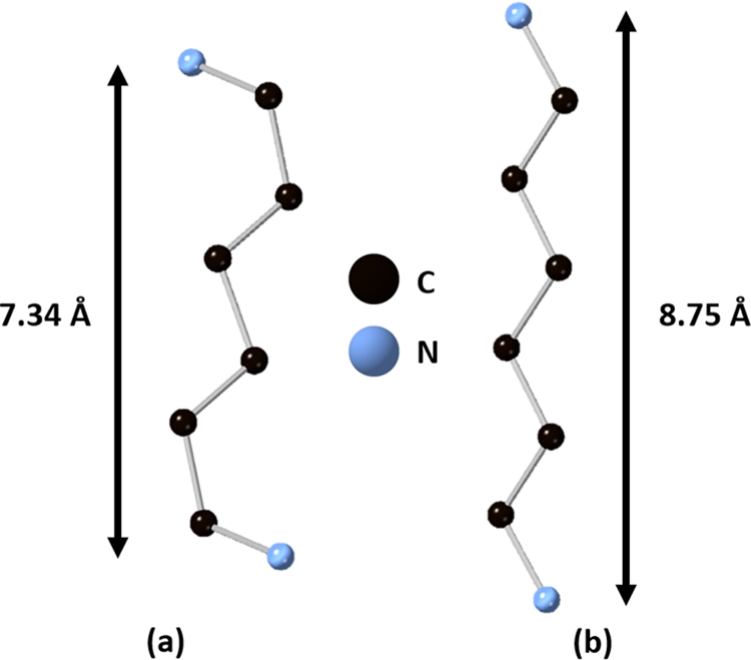
(a, b) Conformations of the H_3_N(CH_2_)_6_NH_3_^2+^ cation of [H_3_N(CH_2_)_6_NH_3_]PbBr_4_ (phase 1) and [H_3_N(CH_2_)_6_NH_3_]PbBr_4_·Br_2_, respectively, obtained from single-crystal X-ray diffraction data.

As shown in Figure 4, the carbon chain of the amine in [H_3_N(CH_2_)_6_NH_3_]PbBr_4_ exhibits some folding in its conformation (which can be described as having a conformation of gtttg, where g = gauche and t = trans), whereas the carbon chain in [H_3_N(CH_2_)_6_NH_3_]PbBr_4_·Br_2_ has an all-trans (i.e., fully stretched) conformation. We also note that the four distinct amine chains in the complex RT phase (phase 3) of [H_3_N(CH_2_)_6_NH_3_]PbBr_4_ all adopt this conformation. Such differences in conformation for differing chain lengths is common in homologous layered perovskite series with differing number of carbons.^61^ In the present case, our crystallographic studies show that when bromine molecules are introduced into the perovskite, a significant portion of interlayer space is occupied by the halogen molecule. Hence, it can be postulated that this steric effect will reduce the degrees of freedom available to the diammonium cations and encourage the carbon chains to adopt an all-trans conformation (i.e., become fully staggered). In other words, Br_2_ acts like a kind of “comb” in straightening out the amine chains. The distortion of the PbBr_6_ octahedra can be calculated in terms of the bond length distortion and bond angle variance, which was originally introduced by Robinson et al.^62,1^ Both the bond length distortion and bond angle variance are significantly reduced upon bromine intercalation (Table 2). The low-temperature structures show that the bond length distortion decreases from 55.63 × 10^–6^ to 0.47 × 10^–6^ upon intercalation, while the bond angle variance decreases from 6.13 to 3.43 upon intercalation. This also means that the octahedral distortion is smaller when the layer shift is smaller. Moreover, the absolute values of the Pb–Br bond lengths in [H_3_N(CH_2_)_6_NH_3_]PbBr_4_ (2.9722(5)–3.0197(4) Å) are reduced slightly to an average of 2.965 Å after the Br_2_ molecules are intercalated. We note that the photoluminescence exhibited by organic–inorganic metal halides has been shown to be strongly dependent on the degree of octahedral distortion.^63^

**Table 2:** Pb-–Br Bond Lengths, Angles, Bond Length Distortions (Δd) and Bond Angle Variances (σ^2^) obtained for [H_3_N(CH_2_)_6_NH_3_]PbBr_4_ and [H_3_N(CH_2_)_6_NH_3_]PbBr_4_·Br_2_ Phases

	[H_3_N(CH_2_)_6_NH_3_]PbBr_4_·Br_2_ LT Pb1	[H_3_N(CH_2_)_6_NH_3_]PbBr_4_·Br_2_ RT Pb1	[H_3_N(CH_2_)_6_NH_3_]PbBr_4_ phase 1 Pb1
Pb–Br (Å)	2.9623(8)	2.9665(8)	2.9722(5)
	2.9623(8)	2.9666(8)	2.9723(5)
	2.9640(7)	2.9771(8)	3.0178(4)
	2.9640(7)	2.9771(8)	3.0178(4)
	2.9672(8)	2.9790(8)	3.0197(4)
	2.9672(8)	2.9790(8)	3.0197(4)
Br–Pb–Br range (deg)	87.247(9)–92.753(9)	87.332(10)–92.667(10)	86.562(12)–93.438(12)
Pb–Br–Pb (deg)	180.0 (×3)	180.0 (×3)	180.0 (×3)
Δ*d* (×10^–6^)	0.47	3.39	55.63
σ^2^	3.43	3.35	6.13

**Figure 5:**
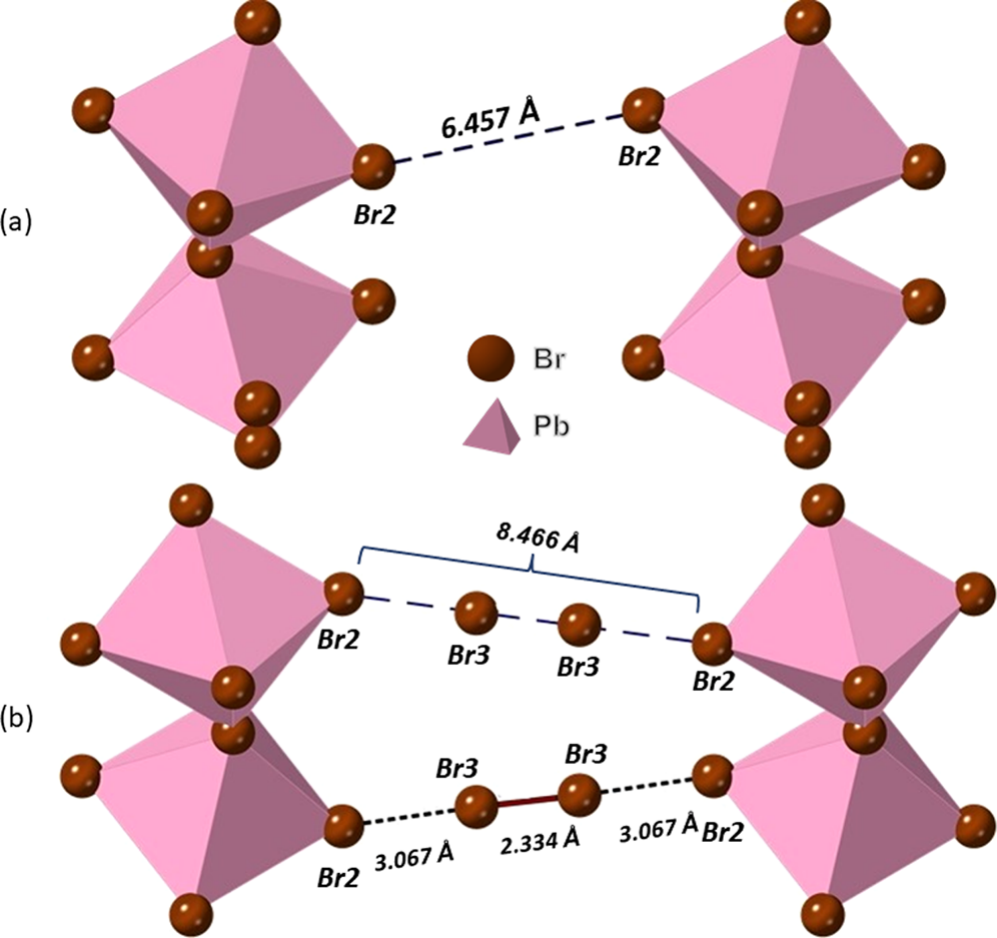
Details of the interlayer interactions of the inorganic components of (a) [H_3_N(CH_2_)_6_NH_3_]PbBr_4_ (phase 1) and (b) [H_3_N(CH_2_)_6_NH_3_]PbBr_4_·Br_2_ obtained from single-crystal X-ray diffraction data.

The position of the bromine molecule, and its interaction with the perovskite layers, can explain how the unusual stability of this intercalate arises. Careful examination of the crystal structure (Figure 5) shows that the bromine molecules adopt an almost linear orientation relative to the apical bromide ligands of adjacent inorganic layers. The angle Br2···Br3···Br3 is 177.5(3)°, and in addition, there is a slight expansion (0.06 Å) of the Br–Br bond in the intercalated Br_2_ molecule from 2.28 Å (expected in solid state Br_2_) to 2.33 Å.^64^ Both the increase in Br_2_ bond length and the fact that the R–X···B bond angles are close to 180° are signatures of halogen bonding between Br_2_ and [PbBr_4_]_∞_ layers. Three key requirements of halogen bonding are R–X···B bond angles close to 180°, an increase in the R–X bond length, and a X···B distance shorter than the sum of the van der Waal’s radii. Here, the Br···[PbBr_4_]_∞_ layer distance is 3.0664(13) Å and the van der Waal’s radius of Br has been calculated to be 1.83 Å; therefore, twice the Van der Waal’s radius would be 3.66 Å.^65^ This is much less than the Br···[PbBr_4_]_∞_ layer distance that would be expected and is therefore an indication of a halogen bond. Knight et al. recently revisited the crystal structure of the 1,4 dioxane–bromine complex, which contains some of the first crystallographically characterized halogen bonds, and found that the Br_2_ bond length varied from 2.326(2) Å at 4.2 K and 2.341(2) Å at 88 K, which are directly comparable to the Br–Br distances reported here in [H_3_N(CH_2_)_6_NH_3_]PbBr_4_·Br_2_, and are greater than the Br–Br distance in solid or gaseous Br_2_.^64,66^ In the study on the 1,4 dioxane–bromine complex, the difference in the O···Br distance in the crystal structure compared to the sum of van der Waal’s radii is around 0.69 Å, while in [H_3_N(CH_2_)_6_NH_3_]PbBr_4_·Br_2_, the difference in the Br···[PbBr_4_]_∞_ layer distance in the crystal structure compared to the Van der Waal’s radii is 0.593 Å.^65^ We also note that in FA_2_PtI_6_·2I_2_ (where FA = formamidinium), the distance between the iodine and PtI_6_ octahedra is 3.297 Å, while the I–I bond length is 2.773 Å.^40^

We believe that another factor that drives the unusual stability of [H_3_N(CH_2_)_6_NH_3_]PbBr_4_·Br_2_ is the position of the bromine molecule, which means that the intercalated bromine is an almost “perfect fit” in the space available for intercalation. The deintercalation of bromine molecules requires overcoming van der Waal’s forces between bromine and both the diammonium cation and the inorganic framework.

Raman spectroscopy was also used to probe the differences between [H_3_N(CH_2_)_6_NH_3_]PbBr_4_ and [H_3_N(CH_2_)_6_NH_3_]PbBr_4_·Br_2_ at low wavenumbers (Figure S8). [H_3_N(CH_2_)_6_NH_3_]PbBr_4_ exhibited two strong Raman bands at 38 and 73 cm^–1^ and a weaker feature at 122 cm^–1^. Upon intercalation in [H_3_N(CH_2_)_6_NH_3_]PbBr_4_·Br_2_, new bands appeared in the Raman spectra at 52, 65, 82, and 125 cm^–1^, which are in comparable positions to those observed in solid bromine.^67,68^ The peak at 38 cm^–1^ in [H_3_N(CH_2_)_6_NH_3_]PbBr_4_ shifts to higher energies of 32 cm^–1^. Also, it is worth pointing out that in FA_2_PtI_6_·I_2_, a peak at 110 cm^–1^ was attributed to be a signature of an extended polyiodide network, so it is possible that the increased intensity of the peak at 125 cm^–1^ in [H_3_N(CH_2_)_6_NH_3_]PbBr_4_·Br_2_ could be attributed to a polybromide network.^40^

In order to probe the differences in [H_3_N(CH_2_)_6_NH_3_]PbBr_4_ and [H_3_N(CH_2_)_6_NH_3_]PbBr_4_·Br_2_ in more detail, we turned to electronic structure calculations.

### 4.2 Computational Results: Electronic Structure Discussion

**Figure 6:**
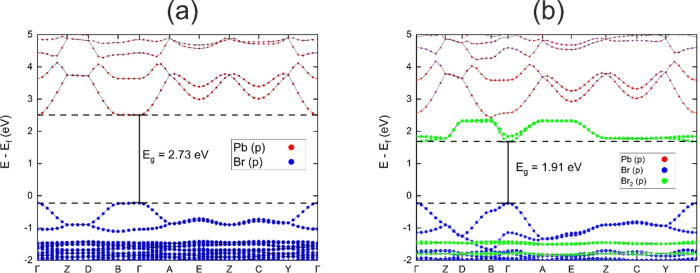
HSE06+SOC projected electron energy band structure of (a) [H_3_N(CH_2_)_6_NH_3_]PbBr_4_ and (b) [H_3_N(CH_2_)_6_NH_3_]PbBr_4_·Br_2_. Projection of the p orbitals of bromine in the octahedra are labeled as Br (p) and the projection of Br_2_ molecules are labeled as Br_2_ (p).

The projected band structures of [H_3_N(CH_2_)_6_NH_3_]PbBr_4_ (phase 1) and [H_3_N(CH_2_)_6_NH_3_]PbBr_4_·Br_2_ (Figure 6a,b) were calculated using the HSE06 hybrid functional and suggested that the occupied band edge for both structures mainly consisted of the 4p states of Br atoms in the perovskite layer. The unoccupied band edge of [H_3_N(CH_2_)_6_NH_3_]PbBr_4_ is dominated by the Pb s states. As for the unoccupied band edge of [H_3_N(CH_2_)_6_NH_3_]PbBr_4_·Br_2_, a band originally from the intercalated Br_2_ molecules’ antibonding orbital is inserted and leads to a direct band gap decrease from 2.73 to 1.91 eV at the Γ point.

A closer look at the band structure shows that the curvature of the p states of Br atoms in the perovskite layer did not change a lot during the intercalation process except for the Γ to Y direction, which is exactly the out-of-plane direction in real space. This is accompanied with the curve in the Br_2_ antibonding orbital in the same direction (which should be flat in a pure Br_2_ molecule). This led us to assume that there are some interactions between the inserted Br_2_ and the Br atoms in the perovskite layer leading to the electron density changing in the out-of-plane direction (see Figure S9).

**Figure 7:**
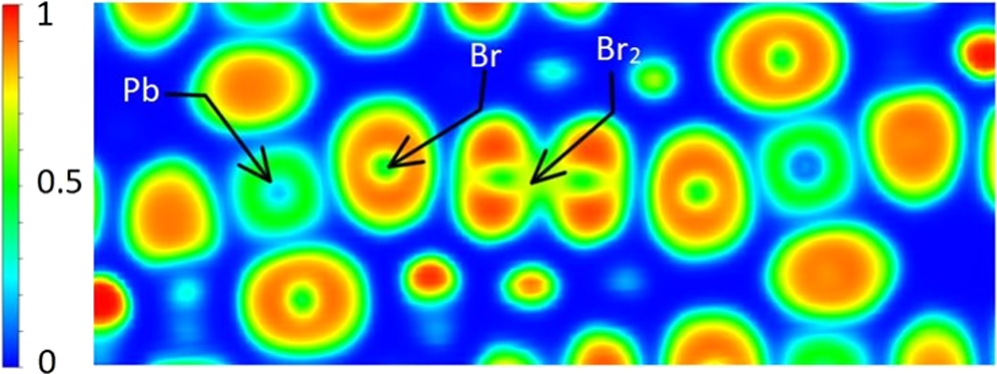
ELF of Br_2_-PbBr_6_ atom chain in [H_3_N(CH_2_)_6_NH_3_]PbBr_4_-Br_2_. The bromine molecule is labeled as Br_2_ and joins two PbBr_6_ octahedra.

In order to understand this interaction between bromine atoms further, we calculated the electron localization function (ELF, Figure 7) along the Br–Pb–Br_2_–Br–Pb atom chain, which was based on the wavefunction generated in the band structure calculation. The yellow contour between the bromine atoms in Br_2_ indicates a strong non-atom-centered electron density, which is evidence of a covalent bond. In contrast, the ELF surrounding the Br atoms in the perovskite layer is atom-centered, indicating that the interactions between Br and Br_2_ molecules are not covalent.

**Figure 8:**
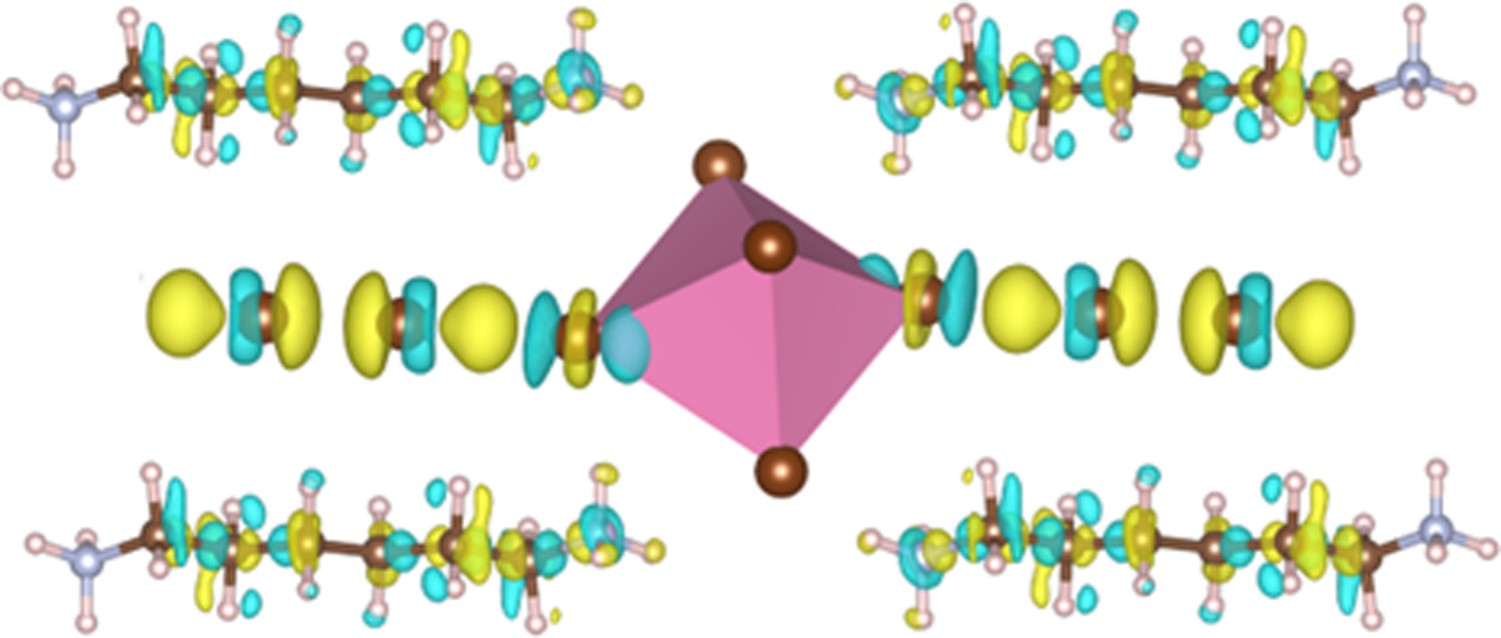
Electron density difference following intercalation of Br_2_ to form the Br_2_-PbBr_6_ atom chain computed using the HSE06+SOC method (the yellow isosurface refers to an increase of electron density by 0.001, cyan to a decrease in electron density by 0.001).

We then compared our results with previous studies on similar structures of X_2_-MX_6_ atom chains connected by halogen bonding, where X refers to halogen atoms and M refers to metals.^39,40^ The halogen bonding effect comes from the anisotropic nature of a strong covalent bond between halogen atoms in a molecule, leading to an electropositive area on the opposite side of the covalent bond called a sigma hole. Such a sigma hole would then attract electronegative atoms such as the halogen atoms in the perovskite layer and help to stabilize the intercalated structure. In order to further probe the presence of the halogen bond in our system, the difference in electron density was calculated for [H_3_N(CH_2_)_6_NH_3_]PbBr_4_·Br_2_ (see Figure 8). The yellow isosurface shows a positive electron density difference on the opposite side of the Br_2_ covalent bond and indicates that the increase in electron density is due to the attraction of the sigma hole and would not be expected if there was no halogen bonding.

As the electron density difference map has shown an increase in electron density, we calculated the effective masses for [H_3_N(CH_2_)_6_NH_3_]PbBr_4_ and [H_3_N(CH_2_)_6_NH_3_]PbBr_4_·Br_2_ (Table 3). As halogen bonding leads to a change in the electron density, we predicted that the mobility of charge carriers in the out-of-plane direction would also change after intercalation.

**Table 3:** Calculated Effective Masses Using HSE06+SOC in Units of Rest Mass of an Electron m_e_

		in-plane (*x*)	in-plane (*y*)	out-of-plane (*z*)
[H_3_N(CH_2_)_6_NH_3_]PbBr_4_	hole	0.426	0.392	7.663
	electron	0.309	0.298	38.606
[H_3_N(CH_2_)_6_NH_3_]PbBr_4_·Br_2_	hole	0.391	0.351	0.244
	electron	1.988	1.954	0.130

As can be seen by the effective masses in Table 3, upon intercalation, the out-of-plane effective mass significantly decreases by around two orders of magnitude. The very large effective mass in the out-of-plane direction of [H_3_N(CH_2_)_6_NH_3_]PbBr_4_ is due to the charge transfer barrier caused by the insulating H_3_N(CH_2_)_6_NH_3_^2+^ cation. In [H_3_N(CH_2_)_6_NH_3_]PbBr_4_·Br_2_, the halogen bonding brought about by intercalation of Br_2_ enabled a charge transfer path to be built in the out-of-plane direction and due to the inverse relationship between effective mass and mobility, we expect that this reduction in effective mass will greatly increase the mobility. The increase of the electron effective mass in the in-plane direction after intercalation was also observed (e.g., for an electron in the in-plane (*x*) direction, the effective mass changes from 0.309 to 1.988 *m*_e_). This can be explained by the electronic structure (Figure 6b), which shows that the Br_2_ band is dispersive in the out-of-plane direction but flat in the in-plane direction.

The decrease in effective masses for both holes and electrons in [H_3_N(CH_2_)_6_NH_3_]PbBr_4_·Br_2_ compared to [H_3_N(CH_2_)_6_NH_3_]PbBr_4_ is particularly significant in the out-of-plane direction and is a decrease of approximately two orders of magnitude. The reduced masses for CH_3_NH_3_PbI_3_ and CH_3_NH_3_PbBr_3_ were reported by Miura et al. and found to be 0.15 and 0.13 m_0_, respectively.^69^ Values for FAPbI_3_ and FAPbBr_3_ have also been reported recently and are 0.095 and 0.13 m_e_, respectively.^30^ The use of experimental spectroscopic techniques in a magnetic field found good agreement to values obtained from computational studies. The in-plane effective masses (i.e., along the PbBr_6_ layers) are comparable to the values obtained for three-dimensional materials such as CH_3_NH_3_PbI_3_ and FAPbI_3_, in addition to two-dimensional materials such as (BA)_2_(MA)_*n*−1_Ge*_n_*I_3*n*+1_, (BA)_2_(MA)_*n*−1_Sn_*n*_I_3*n*+1_, (BA)_2_(FA)_*n*−1_Sn_*n*_I_3*n*+1_, and Cs_2_PbI_2_Cl_2_.^29,69−72^ DFT studies on the all-inorganic RP phase, Cs_2_PbI_2_Cl_2_, have found effective masses of m*_e/h_ of 0.257/0.531 m_0_ for the in-plane direction and m*_e/h_ of 19.254/7.511 m_0_ for the out-of-plane direction, which is significantly higher than the values we have calculated here for [H_3_N(CH_2_)_6_NH_3_]PbBr_4_·Br_2_.^72^ Due to the relationship between mobility and effective mass, these results suggest that the mobility in [H_3_N(CH_2_)_6_NH_3_]PbBr_4_ will be significantly enhanced through Br_2_ intercalation.

### 4.3 UV–Visible Spectroscopy

**Figure 9:**
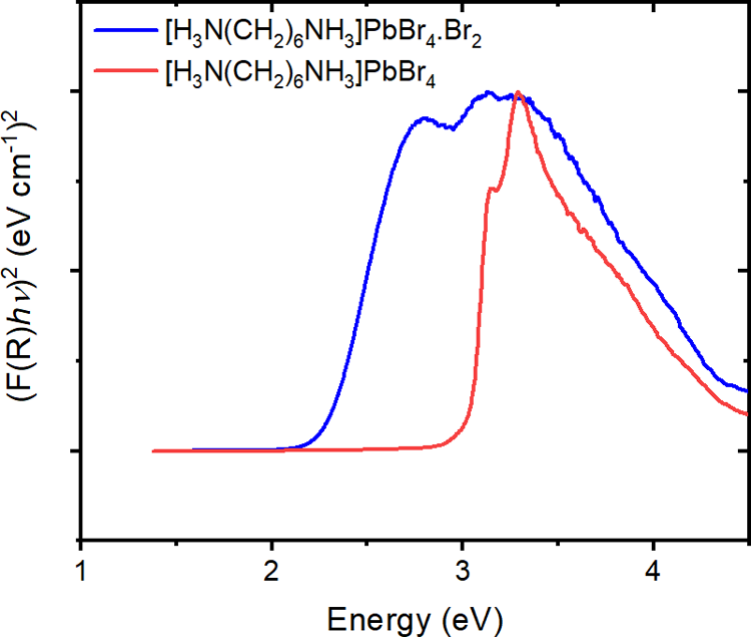
Diffuse reflectance spectra of [H_3_N(CH_2_)_6_NH_3_]PbBr_4_ and [H_3_N(CH_2_)_6_NH_3_]PbBr_4_·Br_2_ plotted using the Kubelka–Munk transformation.

UV–visible spectroscopy was used to determine the band gap of polycrystalline samples of [H_3_N(CH_2_)_6_NH_3_]PbBr_4_ and [H_3_N(CH_2_)_6_NH_3_]PbBr_4_·Br_2_ (Figure 9). The corresponding Tauc plot (Figure 9) was used to determine the band gap. The absorption onset of [H_3_N(CH_2_)_6_NH_3_]PbBr_4_·Br_2_ is red-shifted with respect to [H_3_N(CH_2_)_6_NH_3_]PbBr_4_. The band gap of [H_3_N(CH_2_)_6_NH_3_]PbBr_4_ was found to be 3.00 eV and the band gap of [H_3_N(CH_2_)_6_NH_3_]PbBr_4_·Br_2_ was found to be 2.15 eV. This significant shift in the band gap of 0.85 eV was brought about by the intercalation of bromine in [H_3_N(CH_2_)_6_NH_3_]PbBr_4_ and is in good agreement with the electronic structure calculations (Figure 6).

Interestingly, UV–visible data show a number of clear differences in addition to the decrease in band gap caused by bromine inclusion. The [H_3_N(CH_2_)_6_NH_3_]PbBr_4_ compound shows clear excitonic peaks in the absorption spectra, which are lost upon intercalation, due to the more three-dimensional nature of [H_3_N(CH_2_)_6_NH_3_]PbBr_4_·Br_2_ with respect to [H_3_N(CH_2_)_6_NH_3_]PbBr_4_. [H_3_N(CH_2_)_6_NH_3_]PbBr_4_·Br_2_ has Br_2_ molecules linking the lead bromide layers through halogen bonding between [PbBr_4_]_∞_ and Br_2_. Therefore, this suggests that the exciton confinement is greater in [H_3_N(CH_2_)_6_NH_3_]PbBr_4_ and the intercalation of Br_2_ will reduce the exciton confinement in [H_3_N(CH_2_)_6_NH_3_]PbBr_4_·Br_2_. This is supported by the fact that the electron density difference map has shown an increase in electron density (due to halogen bonding) and the calculated effective masses are reduced by around two orders of magnitude on going from [H_3_N(CH_2_)_6_NH_3_]PbBr_4_ to [H_3_N(CH_2_)_6_NH_3_]PbBr_4_·Br_2_ (Table 3).

It is notable that the shift in the band gap is larger than that reported by Karundasa for iodine inclusion in (IC_6_H_12_NH_3_)_2_PbI_4_.^38^ It is likely that the use of a diamine in this work could play an important role due to the rigidity of the interlayer spaces imposed by the use of a single diammonium cation instead of two monoammonium cations. In addition, the shift of the inorganic layers with respect to each other, which drives the structure from RP-like to DJ-like is also likely to change the band gap by a small amount. The shift in inorganic layers with respect to one another has been seen in perovskites that utilize aromatic cations and resulted in a small change in band gap.^22^ We note that Nag et al. did not determine the change in band gap for (BA)_2_PbI_4_ and (BA)_2_Pb_4_·I_2_, but based on the data presented, it appears that there was a significant shift in band gap.^37^ The magnitude of band gap shift on going from [H_3_N(CH_2_)_6_NH_3_]PbBr_4_ to [H_3_N(CH_2_)_6_NH_3_]PbBr_4_·Br_2_ is comparable to the 0.75 eV shift observed during halide substitution on going from FAPbI_3_ to FAPbBr_3_.^14^ Therefore, intercalation of halogen molecules can offer an alternative method to tune the band gap of organic–inorganic metal halides.

### 4.4 Resistivity Measurements

**Figure 10:**
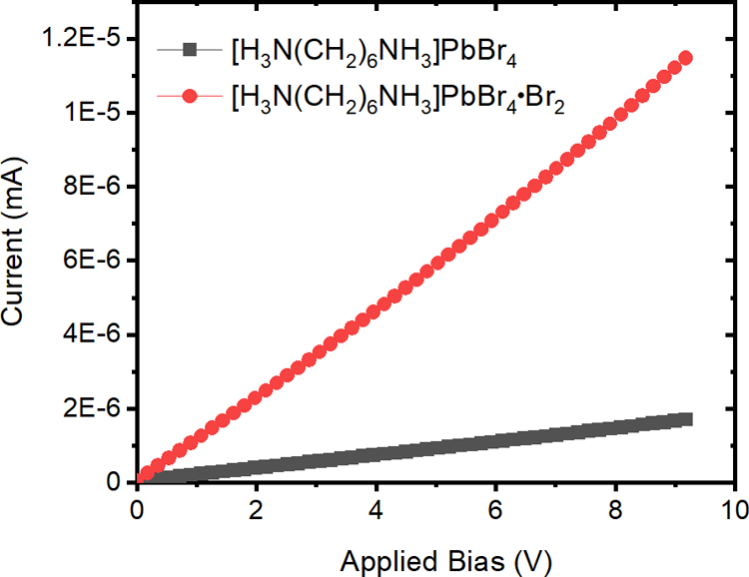
Current–voltage plots of [H_3_N(CH_2_)_6_NH_3_]PbBr_4_ and [H_3_N(CH_2_)_6_NH_3_]PbBr_4_·Br_2_ at room temperature.

In order to study how Br_2_ intercalation changes the properties of [H_3_N(CH_2_)_6_NH_3_]PbBr_4_, the resistivities of [H_3_N(CH_2_)_6_NH_3_]PbBr_4_ and [H_3_N(CH_2_)_6_NH_3_]PbBr_4_·Br_2_ were determined using a polycrystalline sample pressed into a pellet. Figure 10 shows the current–voltage responses from the pellet samples of [H_3_N(CH_2_)_6_NH_3_]PbBr_4_ and [H_3_N(CH_2_)_6_NH_3_]PbBr_4_·Br_2_. As can be seen in Figure 10, the current obtained for both [H_3_N(CH_2_)_6_NH_3_]PbBr_4_·Br_2_ and [H_3_N(CH_2_)_6_NH_3_]PbBr_4_ increases linearly with applied voltage. At room temperature, the resistivity of [H_3_N(CH_2_)_6_NH_3_]PbBr_4_·Br_2_ is 7.01 × 10^6^ Ω cm, one order of magnitude lower than that of [H_3_N(CH_2_)_6_NH_3_]PbBr_4_ that was found to be 5.17 × 10^7^ Ω cm. This is a significant decrease in resistivity of one order of magnitude upon bromine intercalation. This decrease in resistivity is in agreement with the decrease in effective masses that have been obtained in our calculations.

The resistivities reported here are comparable to those reported for other organic–inorganic metal halides. For example, the resistivity of MAPbBr_3_ was reported to be 1.7 × 10^7^ Ω cm.^73^ Ruddlesden–Popper phase (PA)_2_(MA)_2_Pb_3_I_10_ (where PA = propylamine) had resistivities of 10^11^ and 10^8^ Ω cm for the cross-plane and layer plane, respectively.^74^ Zero-dimensional materials such as Cs_3_Bi_2_I_9_ have much higher resistivities; for example, Cs_3_Bi_2_I_9_ had resistivities of 4.58 × 10^10^, 7.62 × 10^10^, and 1.24 × 10^10^ Ω cm when measured along different crystallographic directions.^75^

### 4.5 Measurement of Ionization Potential and Fermi Level

**Figure 11:**
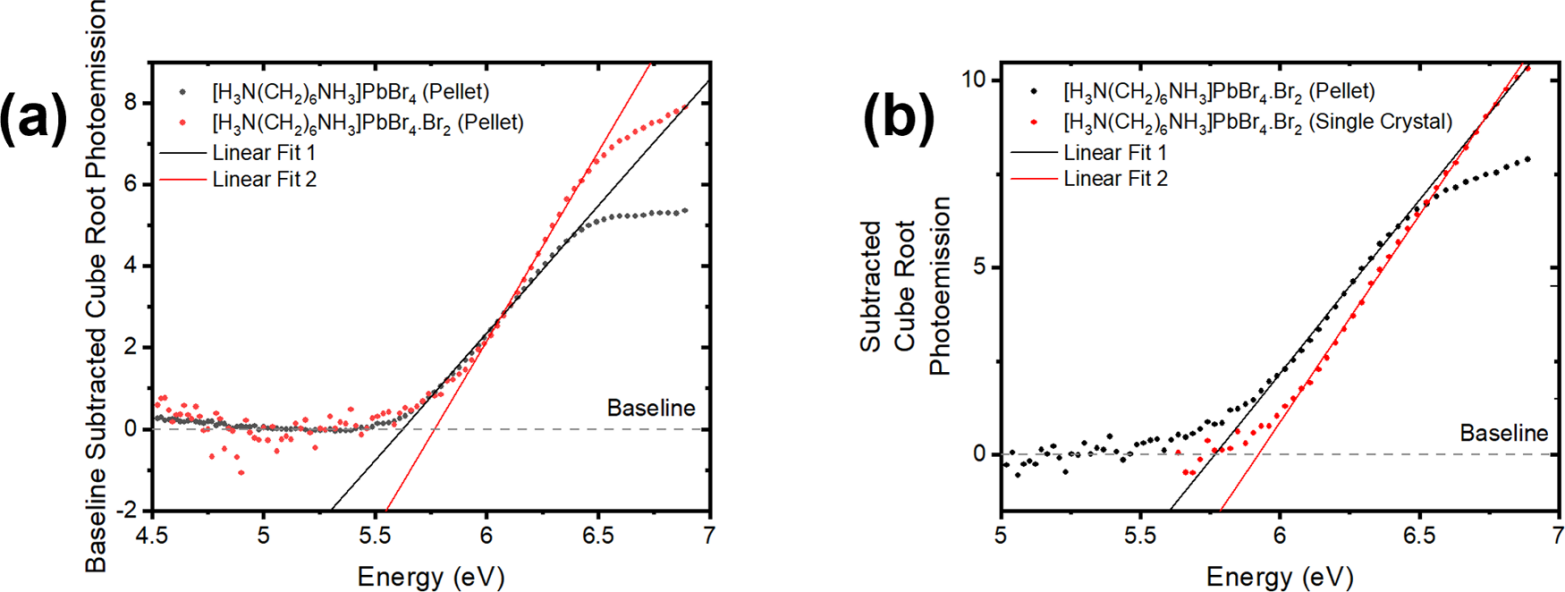
Comparisons of the cube-root of photoemission data for both [H_3_N(CH_2_)_6_NH_3_]PbBr_4_ and [H_3_N(CH_2_)_6_NH_3_]PbBr_4_·Br_2_ samples (a) with baseline-subtracted from each data set. (b) A comparison between the pellet and single-crystal forms of [H_3_N(CH_2_)_6_NH_3_]PbBr_4_·Br_2_ is also shown, indicating that the morphology of the sample also affects the electronic structure of the samples.

To determine the ionization potentials of [H_3_N(CH_2_)_6_NH_3_]PbBr_4_ and [H_3_N(CH_2_)_6_NH_3_]PbBr_4_·Br_2_, ambient-pressure photoemission measurements (APS) were made using the polycrystalline samples pressed into a pellet, and also using a large single crystal of the intercalated perovskite. Figure 11 shows the cube-root of the baseline-subtracted photoemission yields.

The measured ionization potentials of the polycrystalline samples were determined to be 5.62 and 5.76 eV for [H_3_N(CH_2_)_6_NH_3_]PbBr_4_ and [H_3_N(CH_2_)_6_NH_3_]PbBr_4_·Br_2_, respectively. Photoemission from the single crystal of the intercalated perovskite shows an increased ionization potential of 5.98 eV. In all samples, we see evidence for additional energy states at shallower energies than the ionization potential, implying there are some energy states lying within the band gap of the material.

The APS data shows that the intercalation of bromine molecules causes only a small change to the valence band maximum of the perovskite. The change in ionization potential on the intercalation of Br_2_ is much smaller than the 0.85 eV change in band gap from UV–vis measurements, consistent with the computational result that the primary change in the band structure of the perovskites during intercalation is the insertion of an additional band below the conduction band minimum.

**Figure 12:**
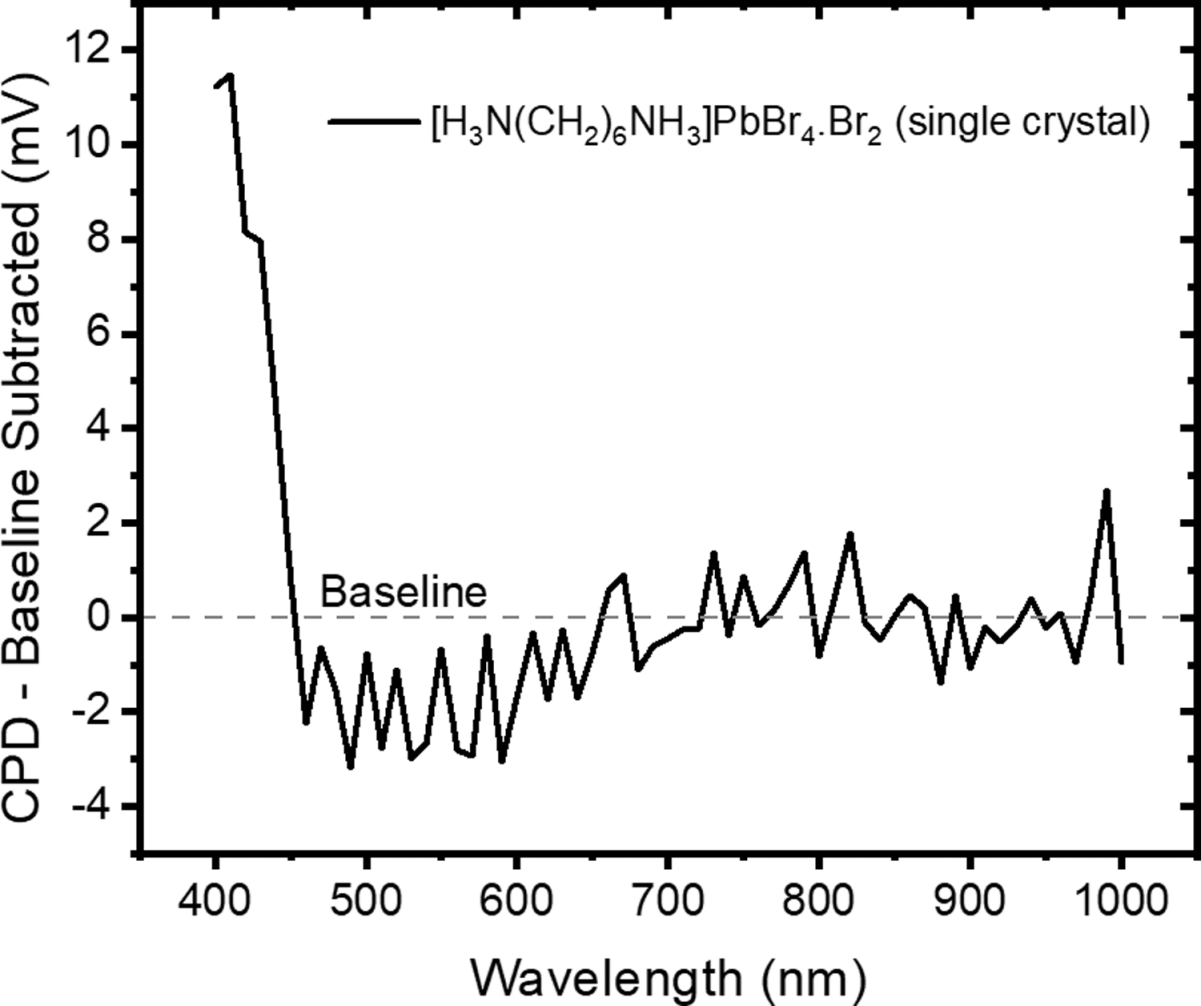
Surface photovoltage spectroscopy measurement of [H_3_N(CH_2_)_6_NH_3_]PbBr_4_·Br_2_ in single-crystal form, showing the measured change in CPD under illumination by different wavelengths of light.

CPD measurements of the same single crystal of the intercalated perovskite found a value of −4.17 eV for the surface Fermi level, lying ∼0.4 eV below the conduction band minimum. Taken with the APS and band gap measurements, this indicates that the material has a natural n-type doping. Figure 12 shows the change in the surface Fermi level when the crystal is illuminated with light. We observe a small surface photovoltage shift toward the conduction band minimum for 450–600 nm excitation, indicative of filling surface trap states when exciting into the new band attributed to the intercalation of Br_2_. For excitation wavelengths below 450 nm, a larger shift toward mid-band gap is observed, possibly due to an increase in the carriers from photogeneration.

### 4.6 Stability Studies

In order to probe the stability of [H_3_N(CH_2_)_6_NH_3_]PbBr_4_·Br_2_, powder X-ray diffraction patterns were collected at room temperature over the course of 10 days (see Figure S7). On day 2, extra peaks appeared in the diffraction pattern that could be indexed to the deintercalated [H_3_N(CH_2_)_6_NH_3_]PbBr_4_ phase 3, but this phase was the minor phase. The phase fraction of the deintercalated [H_3_N(CH_2_)_6_NH_3_]PbBr_4_ increased over the course of the experiment and the PXRD pattern obtained on day 10 showed no evidence of the intercalated material, [H_3_N(CH_2_)_6_NH_3_]PbBr_4_·Br_2_.

**Figure 13:**
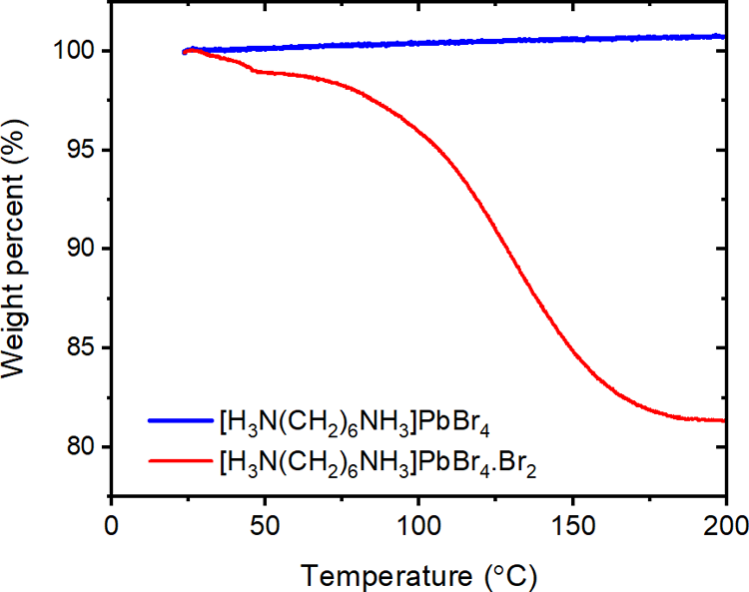
TGA curves for [H_3_N(CH_2_)_6_NH_3_]PbBr_4_ and [H_3_N(CH_2_)_6_NH_3_]PbBr_4_·Br_2_ upon heating from room temperature to 250 °C at 5 °C/min in air.

In order to determine the thermal stability of [H_3_N(CH_2_)_6_NH_3_]PbBr_4_·Br_2_ and [H_3_N(CH_2_)_6_NH_3_]PbBr_4_ phases, thermogravimetric analysis was carried out from room temperature to 200 °C (Figure 13). We can see that the mass loss occurs in two stages for [H_3_N(CH_2_)_6_NH_3_]PbBr_4_·Br_2_. First, a small mass loss is observed between 20 and 50 °C. At 75 °C, [H_3_N(CH_2_)_6_NH_3_]PbBr_4_·Br_2_ begins the second, more significant mass loss, but at the same temperature, no mass loss is observed for [H_3_N(CH_2_)_6_NH_3_]PbBr_4_. At 250 °C, [H_3_N(CH_2_)_6_NH_3_]PbBr_4_·Br_2_ had lost 19.73% mass. This is in good agreement with the expected mass loss of one Br_2_ molecule of 19.9%.

**Figure 14:**
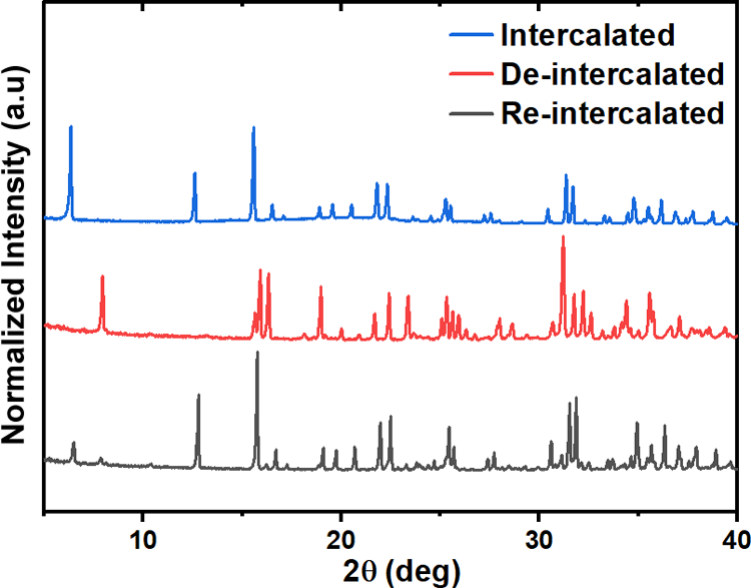
PXRD patterns of [H_3_N(CH_2_)_6_NH_3_]PbBr_4_·Br_2_, [H_3_N(CH_2_)_6_NH_3_]PbBr_4_, and the reintercalated sample.

In order to probe the reversibility of the intercalation process, samples were deintercalated and reintercalated, with the sample purity monitored by PXRD (Figure 14). Samples were reintercalated by exposing the samples to bromine vapor for 5 days in a sealed vessel. As can be seen in Figure 14, the deintercalated sample could be reintercalated, although a small fraction of the deintercalated sample remained in the structure.

## 5 Conclusions

We have shown that we can manipulate the electronic structure and mobility of a layered perovskite through the intercalation of molecular bromine and this offers an alternative way of tuning the band gap in inorganic–organic metal halides. To the best of our knowledge, this is the first example of bromine intercalation in a layered perovskite. Intercalation of bromine results in an increase of the unit cell parameter of 2.36 Å perpendicular to the layer direction, along with a shift from RP-like to DJ-like structures. Electronic structure calculations show that the Br_2_ intercalation results in a new band appearing in the electronic structure. As a result, there is a large decrease of band gap of 0.85 eV. The resistivity of [H_3_N(CH_2_)_6_NH_3_]PbBr_4_·Br_2_ is one order of magnitude smaller than [H_3_N(CH_2_)_6_NH_3_]PbBr_4_, while calculated effective masses indicated a significant decrease along the out-of-plane direction. Together with the calculation of the effective masses, this suggests that bromine intercalation significantly increases the mobility and/or the carrier concentration in [H_3_N(CH_2_)_6_NH_3_]PbBr_4_·Br_2_ in comparison to [H_3_N(CH_2_)_6_NH_3_]PbBr_4_. The intercalation is reversible and also results in a change in the conformation of organic cation, octahedral distortion, and octahedral tilting in the perovskite. By using a combination of computation and crystallography, we have shown that halogen bonding plays an important role in manipulating the structure and properties of [H_3_N(CH_2_)_6_NH_3_]PbBr_4_·Br_2_ and [H_3_N(CH_2_)_6_NH_3_]PbBr_4_, while also offering an alternative method for tuning the electronic properties of organic–inorganic metal halides.

## Data Availability

Data underpinning this work can be found on the University of St Andrews Research Portal: https://doi.org/10.17630/8fe2aa7f-f557-446a-8bbf-c5f33b462ec9.

## Supporting Information Available

Further refinement and crystallographic details, crystal structure diagrams, tables of bond lengths and bond angles, PXRD data, stability studies, Raman spectra, and SEM images (PDF)
