# Supplementary material for: Manipulation of the Structure and Optoelectronic Properties through Bromine Inclusion in a Layered Lead Bromide Perovskite
Source: Chem Mater. 2023 May 3;35(10):3801–14. doi: 10.1021/acs.chemmater.2c03125 (PMC10210243; doi:10.1021/acs.chemmater.2c03125)
Supplement: Supplementary file 1 — cm2c03125_si_001.pdf [file cm2c03125_si_001.pdf]

Supplementary information for

Manipulation of structure and optoelectronic properties through bromine inclusion in a layered lead bromide perovskite

Lin-jie Yang[1], Wenye Xuan[2,3], David Webster[4], Lethy Krishnan Jagadamma[4], Teng Li[1], David N. Miller,[1] David B. Cordes[1], Alexandra M. Z. Slawin[1], Graham A. Turnbull[4] , Ifor D. W. Samuel[4] , Hsin-Yi Tiffany Chen[3], Philip Lightfoot[1], Matthew S. Dyer[2], Julia L. Payne[1*]

[1]*School of Chemistry, University of St Andrews, North Haugh, St Andrews, Fife, KY16 9ST*

[2]*Department of Chemistry, University of Liverpool, Crown St, Liverpool, L69 7ZD and Materials Innovation Factory, University of Liverpool, 51 Oxford St, Liverpool, L7 3NY.*

[3]*Department of Engineering and System Science, National Tsing Hua University, Hsinchu 30013, Taiwan.*

[4]*School of Physics and Astronomy, University of St Andrews, North Haugh, St Andrews, Fife, KY16 9SS*

**jlp8@st-andrews.ac.uk*

Table S 1: Refinement Details of Phase 2 and Phase3 of $[H_3N(CH_2)_6NH_3]PbBr_4$

Sample	$[H_3N(CH_2)_6NH_3]PbBr_4$ Phase 2	$[H_3N(CH_2)_6NH_3]PbBr_4$ Phase 3
CCDC Code	2203749	2203750
Formula	$Pb_3 Br_{12} C_{18} N_6 H_{54}$	$Pb_4 Br_{16} C_{24} N_8 H_{72}$
Formula Weight	645.05	645.05
Crystal Description	Colourless Prism	Colourless Prism
Crystal Size (mm^3)	0.05 x 0.05 x 0.05	0.08 x 0.05 x 0.03
Temperature (K)	93	298
Crystal System	Monoclinic	Monoclinic
Space group	$P2_1$	$P2_1$
a (Å)	16.601(11)	16.382(7)
b (Å)	8.049(4)	8.055(3)
c (Å)	17.329(11)	24.680(10)
β (deg)	97.702(12)	107.187(11)
V (Å^3)	2294.6(2)	3111.3(2)
Z	2	2
ρ_{calc} (g/cm^3)	2.801	2.754
μ (mm^{-1})	21.446	21.090
F(000)	1740	2320
Reflections collected	27314	21917
Independent reflections (R_{int})	8316 (0.0508)	9721 (0.0656)
Parameters, Restraints	221 22	478 2354
Goodness-of-fit on F^2	1.023	0.799
R_1	0.0689	0.1127
R_1 [I>2sigma (I)]	0.0398	0.0520
wR_2	0.0821	0.1438
wR_2 [I>Sigma(I)]	0.0729	0.1210
Largest diff. peak and hole (e/Å^3)	2.132 and -2.255 e.Å^{-3}	1.944 and -2.624 e.Å^{-3}

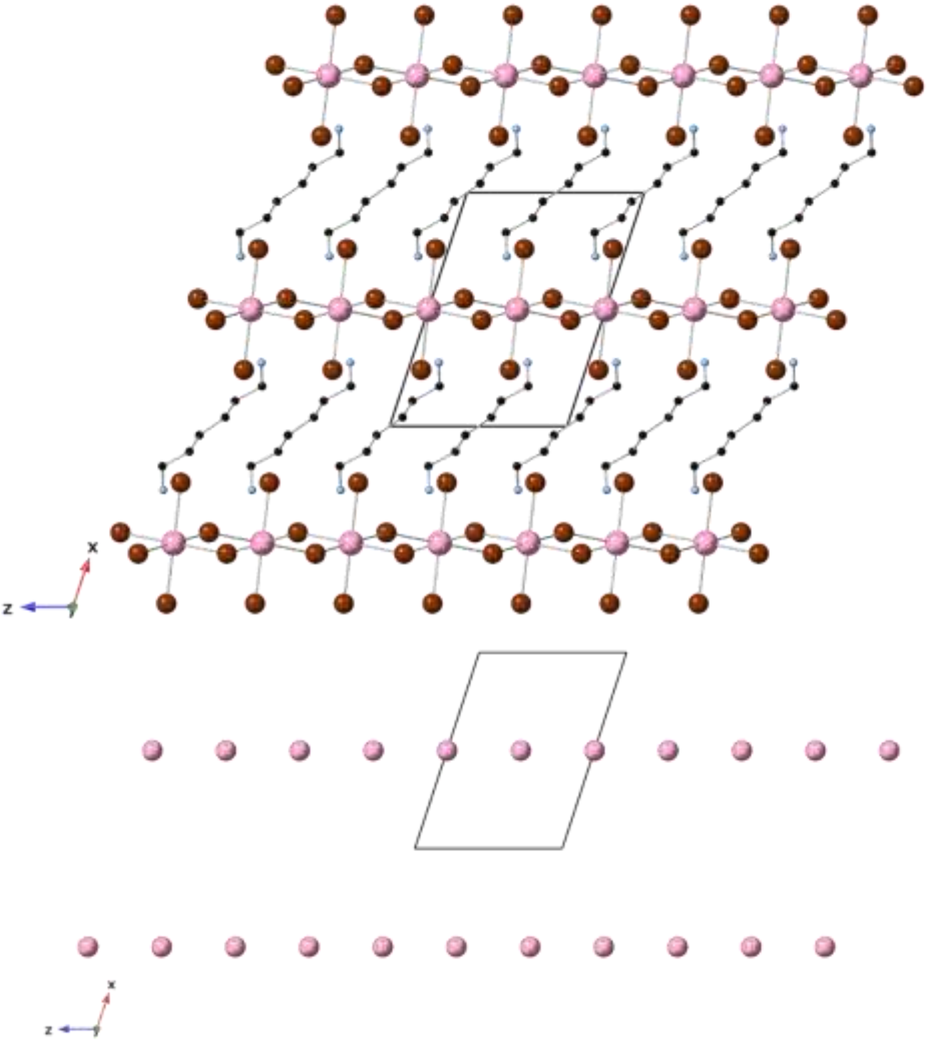

Figure S 1: Phase 1 of $[H_3N(CH_2)_6NH_3]PbBr_4$. The bottom image shows a representation of the Pb atoms only for comparison with Phases 2 and 3. Note the strictly 'flat' Pb layers.

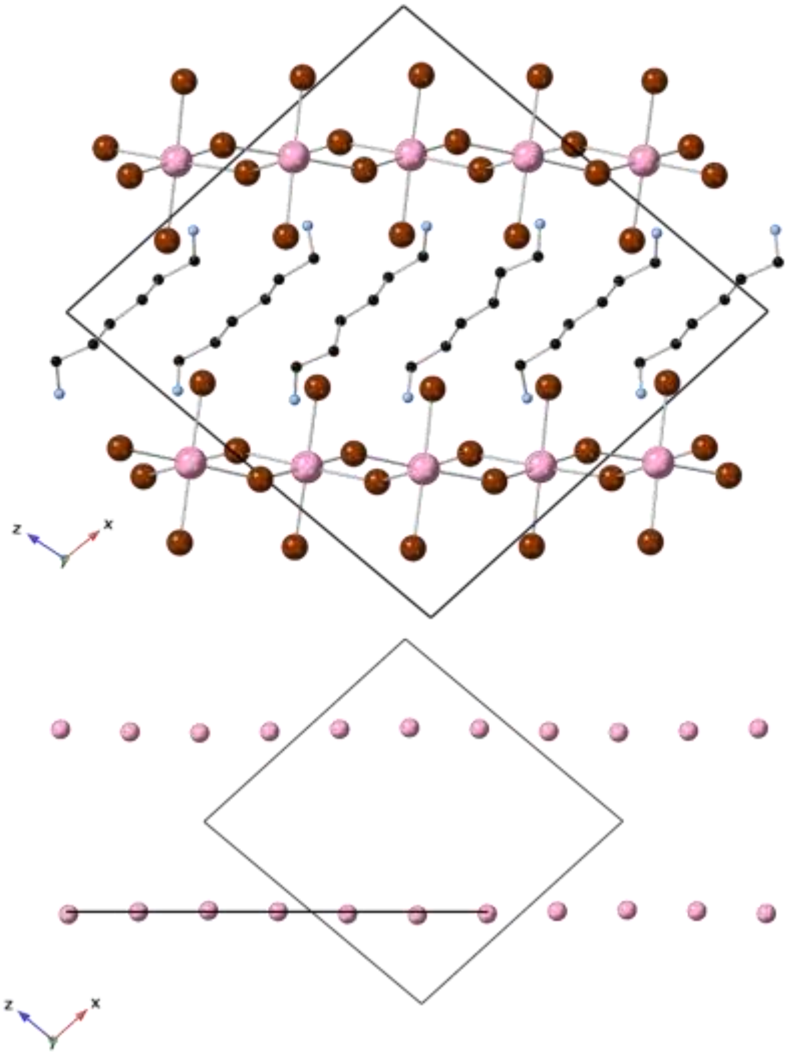

Figure S 2: Phase 2 of $[H_3N(CH_2)_6NH_3]PbBr_4$. Note the very slight undulation of the Pb layers, with a wavelength of six Pb centres.

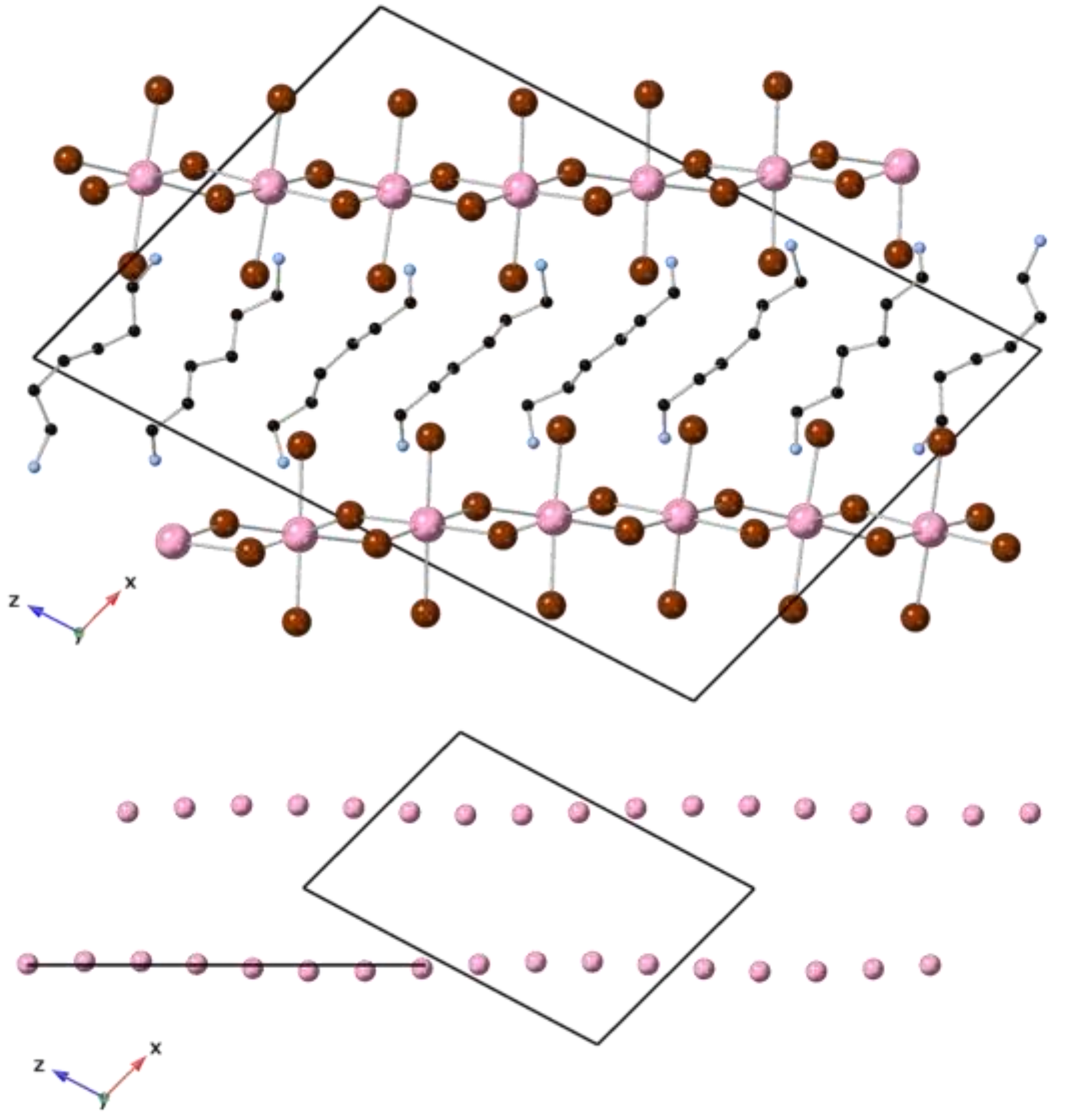

Figure S 3: Phase 3 of $[H_3N(CH_2)_6NH_3]PbBr_4$. Note the more significant undulation of the Pb layers, with a wavelength of eight Pb centres, and also the more significant variations in positioning of the amine moieties.

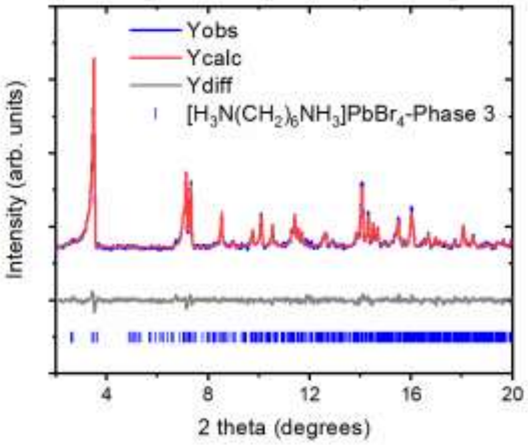

Figure S 4: Rietveld fit of laboratory PXRD data, collected at room temperature using MoK$_{\alpha1}$ radiation, before VT-PXRD experiment, which was carried out on [$H_3N(CH_2)_6NH_3$]$PbBr_4$. Structural model for [$H_3N(CH_2)_6NH_3$]$PbBr_4$ Phase 3 was used in the refinement. Unit cell parameters are as follows: *a* = 16.3940(17) Å, *b* = 8.0510(8) Å, *c* = 24.691(2) Å, β = 107.159(7) °.

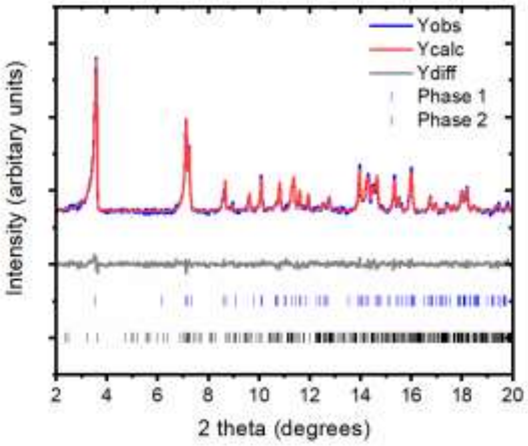

Figure S 5: Rietveld fit of laboratory PXRD data, collected at -50 °C using MoK$_{\alpha1}$ radiation, during VT-PXRD experiment, which was carried out on [$H_3N(CH_2)_6NH_3$]$PbBr_4$. Structural model for [$H_3N(CH_2)_6NH_3$]$PbBr_4$ Phase 1 and Phase 2 was used in the refinement. Unit cell parameters for Phase 1 are *a* = 11.879(5) Å, *b* = 8.047(4) Å, *c* = 8.351(4) Å and β = 105.85(5) ° whilst the unit cell parameters for Phase 2 are *a* = 16.537(2) Å, *b* = 8.0494(8) Å, *c* =17.4063(18) Å and β = 96.715(7) °.

The SEM image of [$H_3N(CH_2)_6NH_3$]$PbBr_4$ is shown in Figure S 6, which shows that [$H_3N(CH_2)_6NH_3$]$PbBr_4$ has adopts a predominantly square prismatic morphology, with dimensions of around 300-400 μm and a thickness of approximately 50 μm. EDS found the Pb: Br ratio to be 20.71 (0.10): 79.29 (0.36), giving a Pb: Br ratio of 1: 3.83, which is within experimental error of the expected 1:4 Pb:Br ratio in the de-intercalated sample.

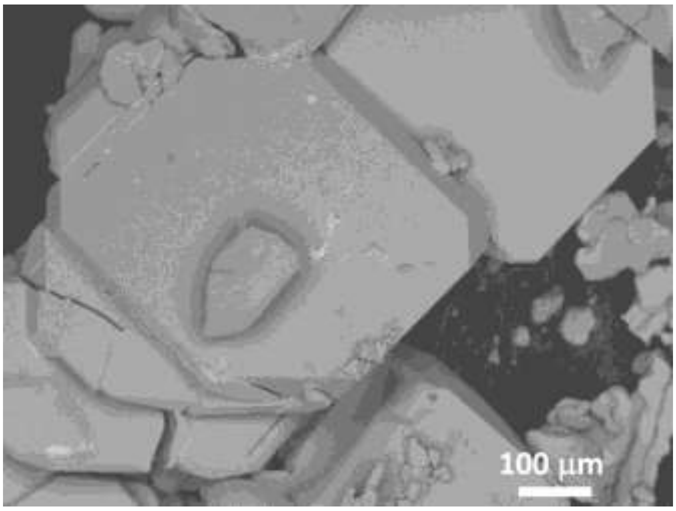

Figure S 6: SEM image of $[H_3N(CH_2)_6NH_3]PbBr_4$

The SEM image of $[H_3N(CH_2)_6NH_3]PbBr_4.Br_2$ is shown in Figure S7. Like $[H_3N(CH_2)_6NH_3]PbBr_4$, the morphology adopted is square prismatic, with dimensions of around 600-800 μm.

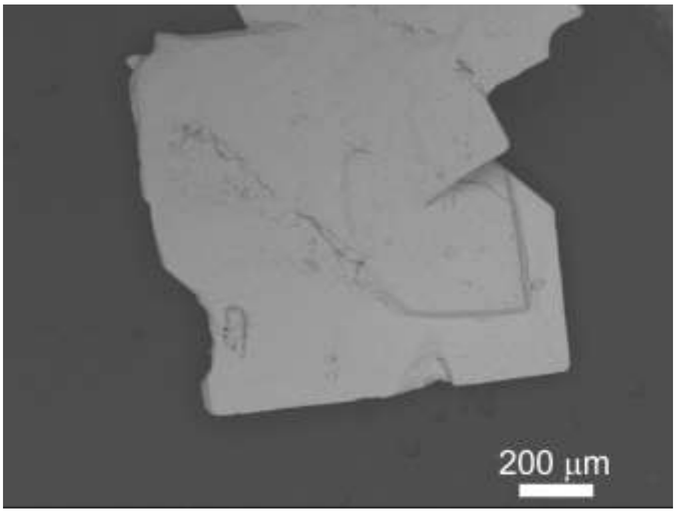

Figure S 7: SEM image of $[H_3N(CH_2)_6NH_3]PbBr_4{\cdot}Br_2$

Table S 1: Pb-Br bond lengths and octahedral distortions for $[H_3N(CH_2)_6NH_3]PbBr_4$ and $[H_3N(CH_2)_6NH_3]PbBr_4.Br_2$

	$[H_3N(CH_2)_6NH_3]PbBr_4.Br_2$ LT	$[H_3N(CH_2)_6NH_3]PbBr_4.Br_2$ RT
	Pb1	Pb1
Pb−Br (Å)	2.9623(8)	2.9665(8)
	2.9623(8)	2.9666(8)
	2.9640(7)	2.9771(8)
	2.9640(7)	2.9771(8)
	2.9672(8)	2.9790(8)
	2.9672(8)	2.9790(8)
Br−Pb−Br range (deg)	87.247(9) - 92.753(9)	87.332(10) - 92.667(10)
Pb−Br−Pb (deg)	180.0	180.0
	180.0	180.0
	180.0	180.0
Δd ($\times 10^{-6}$)	0.47	3.39
σ^2	3.43	3.35

	$[H_3N(CH_2)_6NH_3]PbBr_4$ Phase 2			$[H_3N(CH_2)_6NH_3]PbBr_4$ Phase 3			
	Pb1	Pb2	Pb3	Pb1	Pb2	Pb3	Pb4
Pb−Br (Å)	2.9868(9)	2.9783(10)	2.9937(12)	2.9724(13)	2.9717(14)	2.9957(19)	2.9694(17)
	2.9939(9)	2.9851(10)	3.0073(11)	2.9856(15)	2.9883(15)	3.0048(16)	3.0050(16)
	3.0300(9)	3.0358(10)	3.0324(11)	2.9969(14)	2.9920(17)	3.0063(16)	3.0058(16)
	3.0362(9)	3.0386(10)	3.0352(11)	2.9988(14)	2.9953(15)	3.0091(18)	3.0081(16)
	3.0461(9)	3.0438(10)	3.0357(10)	3.0242(14)	3.0199(14)	3.0118(18)	3.0117(17)
	3.0469(9)	3.0469(10)	3.0510(10)	3.0258(15)	3.0300(14)	3.0126(18)	3.0248(17)
Br−Pb−Br range (deg)	85.63(2) - 94.43(2)	86.30(3) - 94.48(3)	86.16(3) - 94.31(3)	87.18(4) - 93.73(4)	87.07(4) - 93.62(4)	86.64(5) - 93.54(5)	86.76(4) - 93.38(5)
Pb−Br−Pb (deg)	178.60(3)	179.01(3)	179.09(4)	177.03(5)	177.06(5)	179.48(6)	178.85(6)
	178.48(2)	179.20(3)	179.00(3)	178.21(4)	178.36(4)	178.15(6)	178.94(4)
	178.07(2)	178.96(3)	178.82(3)	178.16(4)	177.77(4)	178.26(5)	178.59(5)
Δd ($\times 10^{-6}$)	63.50	88.20	40.70	41.30	42.90	3.53	31.60
σ^2	8.69	9.47	7.44	5.00	5.08	4.82	4.03

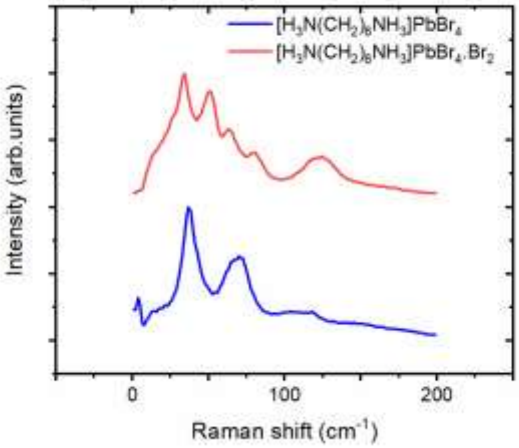

Figure S 8: Raman spectra of $[H_3N(CH_2)_6NH_3]PbBr_4$ and $[H_3N(CH_2)_6NH_3]PbBr_4{\cdot}Br_2$

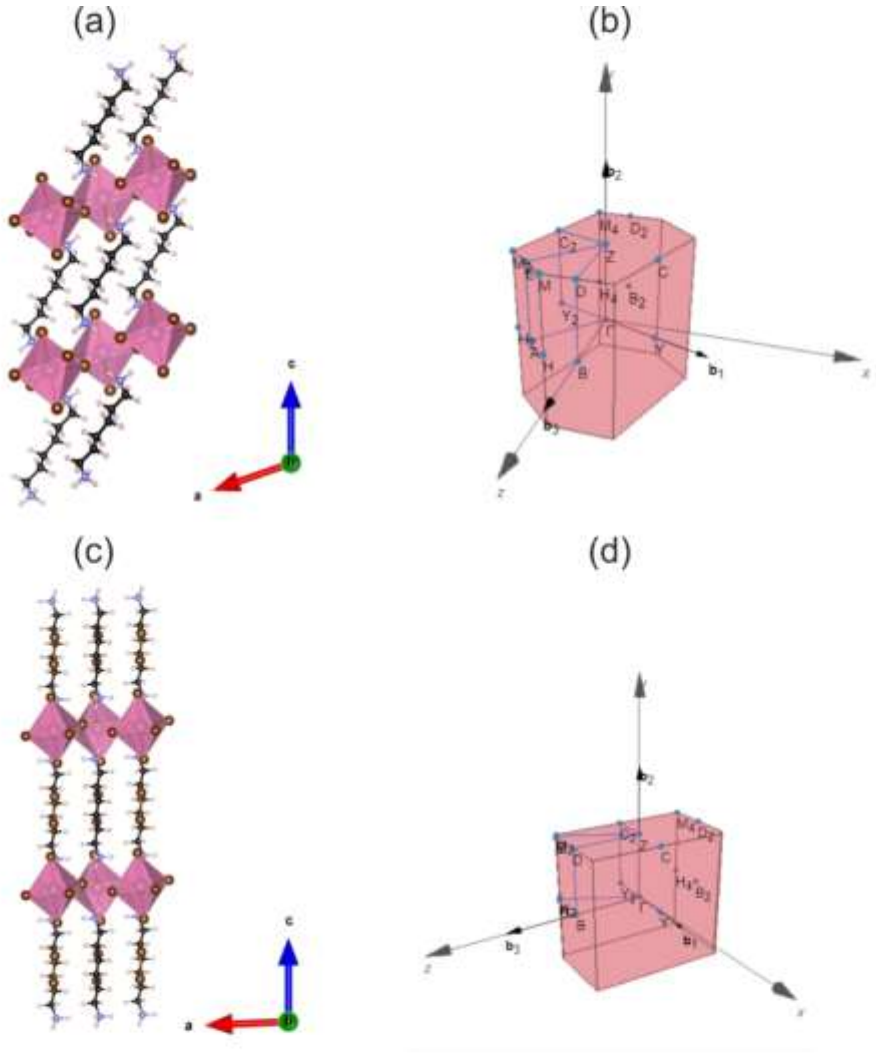

Figure S 9: Computational crystal structure model and Brillouin zone with high symmetry points of $[H_3N(CH_2)_6NH_3]PbBr_4$ (a, b) and $[H_3N(CH_2)_6NH_3]PbBr_4{\cdot}Br_2$ (c, d)

Stability studies

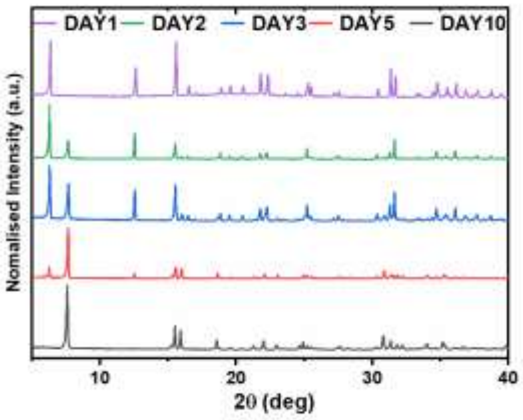

Figure S 10: PXRD patterns of $[H_3N(CH_2)_6NH_3]PbBr_4{\cdot}Br_2$ collected at room temperature over ten days.

In order to probe the stability of $[H_3N(CH_2)_6NH_3]PbBr_4{\cdot}Br_2$, powder X-ray diffraction patterns were collected at room temperature over the course of 10 days (see Figure S10). On day 1, PXRD show that the sample consisted of phase pure $[H_3N(CH_2)_6NH_3]PbBr_4{\cdot}Br_2$, with no evidence of $[H_3N(CH_2)_6NH_3]PbBr_4$. On day 2, extra peaks appeared in the diffraction pattern which could be indexed to the deintercalated $[H_3N(CH_2)_6NH_3]PbBr_4$ Phase 3, but this phase was the minor phase. The phase fraction of $[H_3N(CH_2)_6NH_3]PbBr_4$ continued to increase even further and on day 10 showed no evidence of $[H_3N(CH_2)_6NH_3]PbBr_4{\cdot}Br_2$ at all. The unit cell parameters of $[H_3N(CH_2)_6NH_3]PbBr_4$ and $[H_3N(CH_2)_6NH_3]PbBr_4{\cdot}Br_2$ do not change significantly over time and we have not seen any evidence of ordered phases with bromine and bromine vacancies. In terms of stability, this is better than $(BA)_2PbI_4.I_2$ which full reverted to $(BA)_2PbI_4$ 24hrs after intercalation.[1]

In order to probe the influence of temperature on the long term stability of $[H_3N(CH_2)_6NH_3]PbBr_4{\cdot}Br_2$, samples of $[H_3N(CH_2)_6NH_3]PbBr_4$. Br_2 were stored in the refrigerator at 5 °C for 21 days. The sample was taken out of the refrigerator in order to collect PXRD data at room temperature. The PXRD data collected on day 1 and after 21 days in the refrigerator is shown in Figure S11. No peaks corresponding to the parent $[H_3N(CH_2)_6NH_3]PbBr_4$ material or any other impurities were observed. Therefore, the PXRD studies have shown that the $[H_3N(CH_2)_6NH_3]PbBr_4{\cdot}Br_2$ is stable when kept in the refrigerator for at least 21 days.

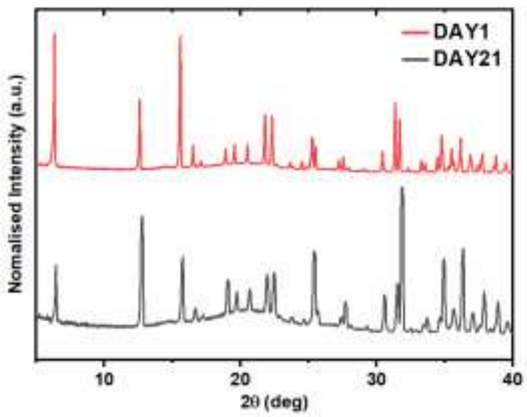

Figure S 11: Powder X-ray diffraction pattern of $[H_3N(CH_2)_6NH_3]PbBr_4{\cdot}Br_2$ after storing for 1 day and 21 days in the refrigerator at approximately 3 to 5 °C

1. T. Sheikh, V. Nawale, N. Pathoor, C. Phadnis, A. Chowdhury and A. Nag, *Angew. Chem. Int. Edit.*, 2020, **59**, 11653-11659.
